# Single-cell copy number variation detection

**DOI:** 10.1186/gb-2011-12-8-r80

**Published:** 2011-08-19

**Authors:** Jiqiu Cheng, Evelyne Vanneste, Peter Konings, Thierry Voet, Joris R Vermeesch, Yves Moreau

**Affiliations:** 1 Department of Electrical Engineering, Esat-SCD, Katholieke Universiteit Leuven, Kasteelpark Arenberg 10, Leuven 3001, Belgium; 2 IBBT-K.U.Leuven Future Health Department, Kasteelpark Arenberg 10, Leuven 3001, Belgium; 3 Center for Human Genetics, Katholieke Universiteit Leuven, Herestraat 49, Leuven 3000, Belgium

## Abstract

Detection of chromosomal aberrations from a single cell by array comparative genomic hybridization (single-cell array CGH), instead of from a population of cells, is an emerging technique. However, such detection is challenging because of the genome artifacts and the DNA amplification process inherent to the single cell approach. Current normalization algorithms result in inaccurate aberration detection for single-cell data. We propose a normalization method based on channel, genome composition and recurrent genome artifact corrections. We demonstrate that the proposed channel clone normalization significantly improves the copy number variation detection in both simulated and real single-cell array CGH data.

## Background

Array analysis of single-cell copy number variations (CNVs) is a recently developed experimental technique for the detection of chromosomal rearrangements in single cells [1-4]. Two-color single-cell array comparative genomic hybridization (CGH) assays the copy number difference between an euploid reference sample from genomic DNA and an unknown test sample from amplified single-cell DNA by comparing signal intensities using log2 ratios [5]. However, the accurate detection of single-cell CNV has been hampered by the noise levels in the log2 ratios caused by the amplification of the minute quantities of DNA present in a single cell. Moreover, since the reference DNA in single-cell array experiments is non-amplified genomic DNA extracted from a large number of cells [2], the biological nature of test and reference sample is different, resulting in new genome artifacts [6]. Unfortunately, existing normalization strategies do not provide clear guidelines for checking for these artifacts, nor for handling them appropriately.

Among existing array CGH normalization methods, global loess normalization is commonly used [7]. Global loess normalization regresses the log2 ratios between test and reference samples on intensities using all probes [8]. The snapCGH package commonly used for analyzing array CGH data has included the global loess normalization method [9]. Furthermore, poplowess and CGHnormaliter have been developed for array CGH data [10,11]. Poplowess attempts to separate normal from aberrant probes using k-means clustering and applies the loess normalization based on the largest group of probes, whereas CGHnormaliter combines a segmentation algorithm with loess normalization iteratively and normalizes data based on segmented normal probes. Although these two methods are supposed to help correctly recognize real chromosomal aberrations, they are not able to correct genome artifacts and could result in false calling of aberrations. Alternatively, the smoothing wave algorithm has been devised to remove genome artifacts that are either related to the GC content or other unknown factors [12]. However, this method requires calibrated genome profiles that are typically not available in the single-cell setup. Recently, more advanced algorithms have been proposed based on the combination of normalization, segmentation, and copy number calling [13-16]. These algorithms allow simultaneous normalization and segmentation and are expected to jointly improve the CNV detection performance. However, these advanced algorithms have been developed for genomic array CGH data and not for single-cell array CGH data, which has an additional artifact-causing property compared to genomic data. All of these normalization methods have in common that they normalize data on the ratio of both channels without taking the single-cell amplification bias and genome artifacts into account.

In this paper, we present a new normalization approach based on channel and clone-specific artifact corrections, named channel clone normalization, to remove the amplification bias caused by the different natures of test and reference samples. Moreover, this approach removes genome artifacts that obscure the detection of real aberrations. The explorations of the amplification bias and genome artifacts are shown in the Results section. Furthermore, we compare our newly developed method to several existing normalization methods (global loess, poplowess, and CGHnormaliter) as well as to the methods combining normalization and segmentation (Haarseg, genome alteration detection analysis (GADA), and circular binary segmentation (CBS) combined normalization) [13,15,16]. The significant performance improvement of our channel-specific normalization method is shown for both simulated and real single-cell array CGH data.

## Results

### Simulation of single-cell data

**Figure 1:**
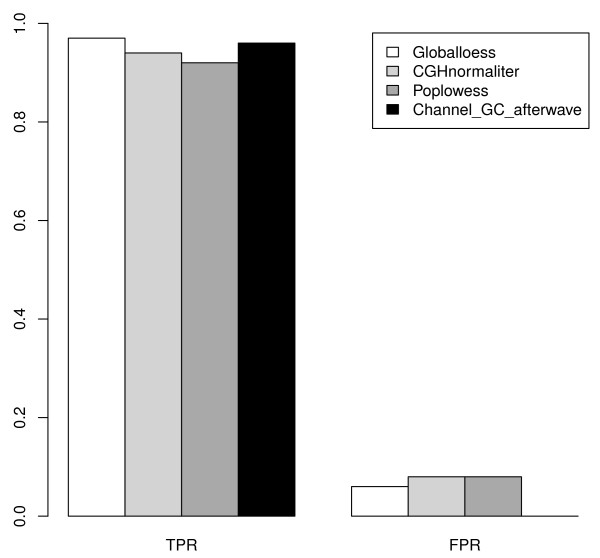
**Barplot of true positive rate and false positive rate of 15 simulated samples**. All the true positive rates (TPRs) and false positive rates (FPRs) were calculated after the global loess, CGHnormaliter, poplowess or channel clone normalization methods.

To quantify the effect of the channel clone normalization, we simulated 15 samples including 23 artificial aberrations based on 7 real Epstein-Barr virus (EBV)-transformed samples as described in the Application section. The simulation details are presented in the Materials and methods section. This simulation data set is comparable to real genome profile features of the single-cell array CGH data with known artificial aberrations. The overall performance of all normalization methods on the simulation data set is demonstrated in Figure 1. The true positive rates (TPRs) using global loess, CGHnormaliter, poplowess, and channel clone normalization are 0.97, 0.94, 0.92, and 0.96, respectively, whereas the false positive rates (FPR) are 0.06, 0.08, 0.08 and 0, respectively. Although channel clone normalization missed 1 out of the 23 known aberrations, it offers the best performance in comparison to the other normalization methods with the fewest falsely discovered CNV regions and comparable TPR. Global loess, CGHnormaliter, and poplowess show similar CNV detection performance in terms of TPR and FPR.

**Figure 2:**
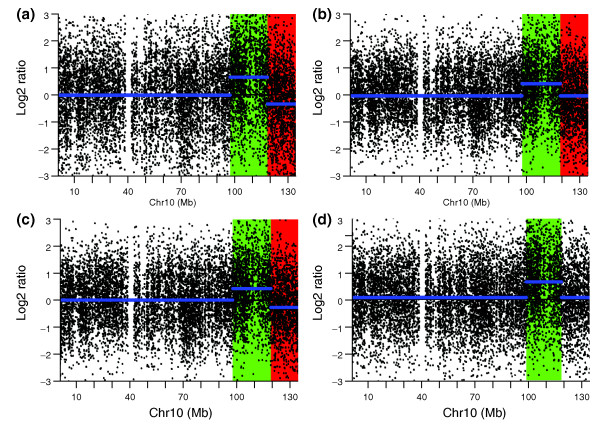
**Copy number variation detection in chromosome 10 of a simulated sample**. (a-d) CBS segmentation of chromosome 10 from the simulated sample 4 using global loess normalization (a), CGHnormaliter (b), poplowess (c), and channel clone normalization (d). The blue line represents the CBS segmentation line. The red region and green regions represent the deletion and duplication regions called by CGHcall.

An example shown in Figure 2 illustrates the correction of genome artifacts by channel clone normalization. Chromosome 10 of sample 4 contains a confirmed duplication on the q-arm. This duplication was correctly detected by all four normalization methods. However, the chromosome 10 q-terminal region was incorrectly detected as a deletion using global loess, CGHnormaliter, and poplowess. In contrast, this genome artifact was corrected by the channel clone normalization method and detected as a normal region.

### Application 1: single EBV-transformed lymphoblastoid cell array CGH

We analyzed seven single EBV-transformed lymphoblastoid cells amplified according to the previously described protocol [2]. Each of these amplified single-cell DNA samples was hybridized as a test sample on Agilent 244 K arrays against genomic non-amplified DNA derived from a patient with Klinefelter syndrome (47, XXY). The aberration and diploid regions have been validated by the corresponding genomic DNA using a 250 K Affymetrix SNP array with the help of SNP copy number, loss-of-heterozygosity, and heterozygous SNPs. The karyotype of each EBV-transformed sample is shown in Table 1. We used this data set to quantify our approach and benchmark our data with other methods.

**Table 1:** True positive rate of each EBV cell followed by different normalizations

Real aberrations^a^	Global loess	CGHnormaliter	Poplowess	Haarseg	CG probeA	CG	CA	CGACBS	Channel clone
Cell 617, 14 M, 18p ter del	0	13.62	13.72	13.56	14.18	14.86	12.05	14.18	11.99
Cell 1151, 9.3 M, 18p, dup	0	0	0	9.04	8.87	6.21	9.06	8.92	8.87
Cell 1151, 1.7 M, 20p ter del	1.70	1.70	1.70	0	1.70	1.70	0	1.70	0
Cell 1151, monosomy X	151.87	151.87	151.87	151.59	151.87	151.87	151.87	151.87	151.87
Cell 1160, 3 M, 11 qter del	2.22	1.73	2.56	0	2.20	2.22	0	2.22	2.56
Cell 1162, 47.5 M, 14q dup	0	0	0	40.14	31.97	45.78	47.39	39.47	47.39
Cell 1162, 58 M, chr X, del	59.94	59.94	59.94	0	59.93	59.93	57.30	59.92	57.30
Cell 1168, trisomy 21	0	0	0	0	0	0	0	0	36.99
TPR	0.66	0.71	0.71	0.66	0.83	0.86	0.86	0.86	0.98

^a^Validated by Affymetrix 250 K array using genomic DNA. The first column represents the true validated aberrations of each EBV cell, followed by the detected aberration length after global loess, CGHnormaliter, poplowess, Haarseg, CGprobeA, CG, CA, CGACBS and channel clone normalization methods. The column with bold numbers shows the detected aberration length and true positive rate after the channel clone normalization. CA, channel standardization followed by recurrent genome artifact correction without CBS segmentation; CG, channel standardization followed by genome composition correction using enlarged window GC contents; CGACBS, channel standardization followed by genome composition correction using enlarged window GC contents and recurrent genome artifact correction with CBS segmentation; CGprobeA, channel standardization followed by genome composition correction using probe GC contents and recurrent genome artifact correction without CBS segmentation; Channel clone, channel standardization followed by genome composition correction using enlarged window GC contents and recurrent genome artifact correction without CBS segmentation.

Our normalization approach mainly consists of three steps: channel standardization, genome composition artifacts correction and recurrent genome artifacts correction. All of these three steps are necessary to improve single-cell CNV detection. The investigation of the single-cell amplification bias is covered in the 'Exploration of the amplification bias' section and the exploration of genome artifacts is covered in the 'Detection of copy number variation' section.

#### Exploration of the amplification bias

We first explored the amplification bias caused by the different natures of the test and reference samples with the help of graphical plots. MA, density, and quantile-quantile (QQ) plots are used to check for potential artifacts before and after normalization. The *y*-axis and *x*-axis of a MA plot represent the log2 ratios and average log2 intensities between two hybridized samples, respectively. The points of a MA plot should be randomly located around zero in the *y*-axis if no large aberrations or artifacts exist in the data. The density plot and QQ plot are graphical techniques to show the similarity between intensity distributions from test and reference samples. If the test sample intensities are distributed similarly to reference intensities, the density plot of two hybridized samples should overlap and the QQ plot should be located along the 45-degree line.

**Figure 3:**
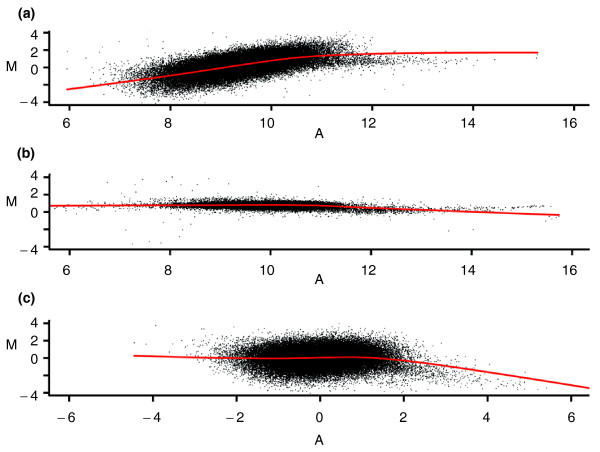
**MA plot of a single EBV-transformed cell**. (a-c) MA plot for EBV-transformed single lymphoblastoid cell 1162 before normalization (a), genomic DNA before normalization (b), EBV-transformed single lymphoblastoid 1162 after channel standardization (c). The red line represents a lowess curve fitted to the data. Note that after normalizations, most of the log2 ratio values are distributed randomly around zero.

**Figure 4:**
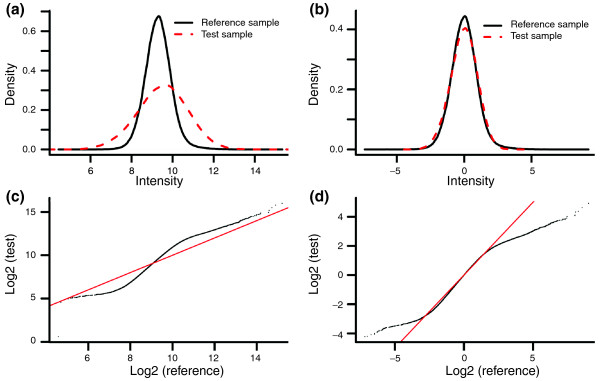
**Density plot of a single EBV-transformed cell**. **(a,b) **Density plot for EBV-transformed single lymphoblastoid cell 1162 before normalization (a), and after channel standardization (b). The solid line represents the reference sample and the dashed line represents the test sample. Note that the SD of the intensities of the test sample (SD = 1.02) is larger than that of the reference sample (SD = 0.61). **(c,d) **QQ plot of the intensities between the test and the reference samples before normalization (c), and after channel standardization (d).

An obvious intensity-dependent pattern is observed in the MA plot of all single-cell array CGH experiments (Figure 3a; Additional file 1). The pattern visualized using the red lowess smoothing line shows that the log2 ratio increases nonlinearly with the increase of the average intensities in the single-cell array CGH data. In contrast, the MA plot of an array CGH experiment using non-amplified genomic DNA shows no aberrant pattern (Figure 3b). Since both array CGH experiments were performed using the same series of Agilent 244K arrays and the only difference between them was the processing of the test samples, we suspect that the intensity-dependent pattern artifact is caused by the amplification of the single-cell DNA. This suspicion is confirmed by the larger standard deviation (SD) of the intensities in the amplified test sample compared to the non-amplified reference sample (Figure 4a). Consequently, the median SD of single-cell array CGH log2 ratios is 1.38, ranging from 0.85 to 1.44 across 7 arrays, whereas that of the genomic array CGH experiments is 0.28, ranging from 0.2 to 0.35 across 6 arrays. This larger SD of log2 ratios in the single-cell array CGH experiments hampers the accurate detection of CNVs at the single-cell level. It is thus necessary to remove this amplification bias.

After the data are normalized by the channel standardization step, the pattern between averaged intensity and log2 ratio disappears and the lowess curve fitted to the data is close to horizontal (Figure 3c). The intensity distributions of the reference and test samples are adjusted to have approximate mean zero and SD equal to 1 (Figure 4b). The QQ plot in Figure 4d shows that most points after the channel clone normalization are located around the 45-degree reference line, meaning that the intensities of normalized test and reference samples follow similar distributions. We conclude that the amplification bias has been successfully removed by the channel standardization step.

#### Detection of copy number variation

After the exploration of the amplification bias, we checked the impact of genome composition artifacts and recurrent genome artifacts on the performance of single-cell CNV detection using the CBS algorithm [17]. Genome composition artifacts, appearing as incorrect chromosomal aberrations, are frequently observed in the array CGH data. These artifacts are illustrated in Figure S2a,b in Additional file 2 with the low log2 ratios of the chromosome 1 p terminus and the chromosome 10 q terminus. Studies have shown that these genome composition artifacts could be caused by GC content as well as other unknown factors [18].

We therefore use a genome composition correction step to correct the artifacts caused by GC content and a recurrent artifact correction step to correct unknown recurrent artifacts. For the genome composition correction step, we considered two possible methods: correction based on the GC content of (1) the probe sequence itself or (2) an enlarged window around the probe. Similarly, for the recurrent genome artifact correction we also considered two methods: (1) CBS segmented residuals followed by the recurrent genome artifact correction and (2) an artifact correction without the CBS segmentation in advance. The details of the channel clone normalization are introduced in the Materials and methods section. We compare our channel clone approach with four sub-methods to show that the combination of channel standardization, genome composition artifact correction and recurrent genome artifact correction together give the best single-cell CNV detection performance: CG (channel plus genome composition correction using enlarged window GC contents); CA (channel plus recurrent genome artifact correction without CBS segmentation); CGprobeA (channel plus genome composition correction using probe GC contents plus recurrent genome artifact correction without CBS segmentation); CGACBS (channel plus genome composition correction using enlarged window GC contents plus CBS segmented residuals followed by recurrent genome artifact correction); channel clone (channel plus genome composition correction using enlarged window GC contents plus recurrent genome artifact correction without CBS segmentation).

**Figure 5:**
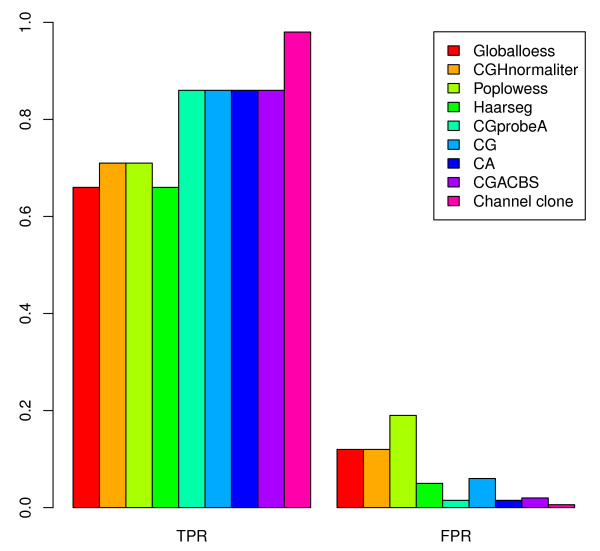
**Barplot of true positive rate and false positive rate of 7 EBV-transformed cells**. All the TPRs and FPRs were calculated after global loess, CGHnormaliter, poplowess, Haarseg, CG, CA, CGprobeA, CGACBS and channel clone normalization approaches.

The genome profiles before and after genome composition correction are shown in Additional file 2. It is obvious that the GC-content-related artifacts, appearing as a wave pattern in Figure S2a,b in Additional file 2 are adjusted after the genome composition correction shown in Figure S2c,d in Additional file 2. Similarly, Figure 5 shows that the CNV detection performance of CG with a TPR 0.86 and FPR 0.06, respectively, is better than for the methods that do not account for genome composition correction (for example, global loess, CGHnormaliter, and poplowess).

Different studies have used genome composition corrections to correct the genome wave pattern [18]. Array CGH hybridization is influenced not only by the GC content of the probe sequence but also the DNA sequences that lie in an enlarged window around the probe sequence corresponding to a DNA sequence fragment the probe hybridizes to. Diskin *et al. *[19] used an ordinary linear regression model to regress the Log2Ratio on the GC content of a fixed 1Mb window size around the probe to correct the genome composition artifacts. Since this method was developed for single-channel arrays and cannot be directly implemented for the two-color arrays, we developed a comparable but more elaborate genome composition correction approach. To account for the GC content of the unknown genome fragments, our method extracts the GC percentage from different window sizes around each probe and elects the window size with the highest correlated GC content to the log2 ratio for the genome composition correction. Secondly, in contrast with Diskin *et al*.'s method, we use a weighted linear regression model with larger weights for the GC-rich probes to avoid the overcorrection of real chromosomal aberrations. Other genome correction methods could also be valid. However, comparison of all GC correction methods is outside the scope of our study. To show that accounting for the GC content from enlarged window sizes improves the genome composition correction, we also performed the correction based only on the GC content of each probe, as proposed by the CGprobeA normalization. Figure 5 shows that the TPR and FPR values are 0.86 and 0.015, respectively, for the CGprobeA normalization method, whereas the values for our channel normalization are 0.98 and 0.006, respectively. This comparison confirms the importance of finding the optimal GC-content window for the genome composition correction.

The impact of the recurrent genome artifact correction of each chromosome is especially explained in Additional file 3 and shown in Additional files 4 to 10. For instance, chromosome 3 of EBV-transformed cell 1168 was experimentally confirmed to have no aberrations. However, two deletions at the location around 50 Mb and the q-arm terminal region were observed when no correction was applied (Figure S3a in Additional file 3). The estimated common profile of chromosome 3 (Figure S3b in Additional file 3) shows the artifacts at the same locations as in the individual profile of EBV-transformed cell 1168. Since the common profile is estimated across all the EBV-transformed samples, the artifacts observed in the common profile represent the recurrent genome artifacts existing in multiple EBV-transformed samples. Figure S3c in Additional file 3 shows that after the extraction of the estimated common profile, these two artifacts have been removed and the segmentation line of this chromosomal profile is horizontal around the zero line.

Comparison of the CG and CA methods to channel clone normalization is shown in Figure 5 andTable 1. Both the CG and CA normalization methods show lower TPRs and larger FPRs for single-cell CNV detection performance. These results confirm our hypothesis that not all genome artifacts can be explained by GC content. Our channel clone normalization method removes genome composition artifacts, as well as unknown recurrent genome artifacts. Therefore, the combination of channel standardization, genome composition and recurrent genome artifact corrections, which we propose, gives the best single-cell CNV detection performance, with a TPR of 0.98 and a FPR of 0.006.

A recent study suggests that the combination of segmentation with recurrent genome artifact correction can improve aberration detection in genomic array CGH applications [16]. We tested this CGACBS approach on our single-cell array CGH data. Table 1 shows that the TPR and FPR of CGACBS are 0.86 and 0.02, respectively, which is outperformed by channel clone normalization, with values of 0.98 and 0.006, respectively. CGACBS uses CBS segmented residuals for genome artifact correction to avoid overcorrection of real chromosomal aberrations. However, this method also protects genome artifacts with log2 ratios comparable to real aberrations from being corrected. Consequently, it results in higher false positive calling of aberrations. Therefore, it is a trade-off between keeping real aberration signals and removing undesired genome artifacts.

Moreover, we have compared our normalization approach to global loess, CGHnormaliter, poplowess, Haarseg, and GADA methods. Using the TPR and FPR as given in Figure 5 andTable 1 we compared the overall CNV detection performance for global loess, CGHnormaliter, poplowess, Haarseg, and channel clone normalization. The TPR values were 0.66, 0.71, 0.71, 0.66, and 0.98, respectively, while the FPRs were 0.13, 0.09, 0.15, 0.05, and 0.006, respectively. Although the recently developed poplowess and CGHnormaliter normalization methods perform better than the original global loess normalization, they have a high FPR as well. The common feature of both methods is the separation of probes with normal log2 ratios from probes with aberrant log2 ratios, as well as the normalization of the data based on the normal probe log2 ratios; however, this is not suitable in single-cell array CGH. The reason is that many genome artifacts appear next to real aberrations caused by amplification bias in the single-cell approach. As a consequence, these genome artifacts are incorrectly segmented or clustered by the CGHnormaliter or poplowess algorithms into aberrant groups, yielding poor results.

The channel clone normalization method has shown its advantage in correcting recurrent genome artifacts across samples. Notice that CBS fails to detect a 2.22 Mb deletion at the chromosome 20 p terminus of cell 1151 after channel clone normalization (Figure S12 in Additional file 12). The possible reason is that this deletion is located in the terminal region of a chromosome with a short length of 2.22 Mb. This aberration thus shows a pattern similar to the artifacts located at the same position and results in an overcorrection by the channel clone normalization. However, considering the large FPR caused by chromosomal artifacts from the single-cell array CGH, it is worthwhile to reduce the FPR from around 10% to 0.6%, even while missing one short aberration.

**Figure 6:**
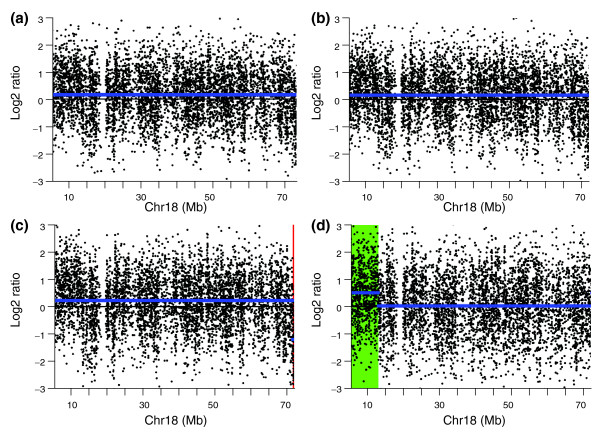
**Copy number variation detection in chromosome 18 of an EBV-transformed sample**. **(a-d) **CBS segmentation of chromosome 18 from the EBV-transformed single lymphoblastoid cell 1151 using global loess normalization (a), CGHnormaliter (b), poplowess (c), and channel clone normalization (d). The *y*-axis represents the log2 ratios and the *x*-axis represents the coordinates along the chromosome. The blue line represents the CBS segmentation line. The green region represents the duplication region called by the CGHcall program.

**Figure 7:**
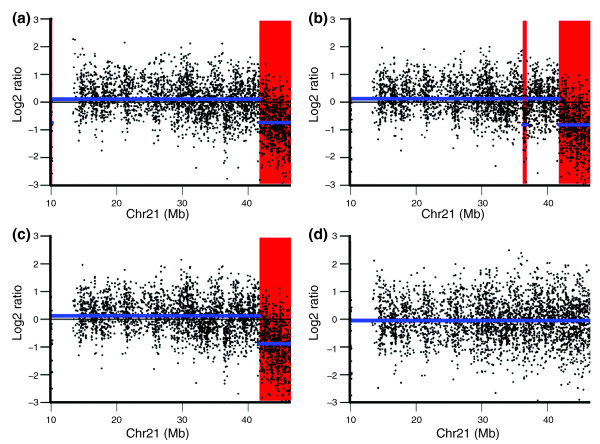
**Copy number variation detection in chromosome 21 of an EBV-transformed sample**. **(a-d) **CBS segmentation of chromosome 21 from the EBV-transformed single lymphoblastoid cell 1160 using global loess normalization (a), CGHnormaliter (b), poplowess (c), and channel clone normalization (d). The blue line represents the CBS segmentation line. The red region represents the deletion region called by CGHcall.

The performance of global loess, CGHnormaliter, poplowess, Haarseg and channel clone normalization on each genome profile is shown in Figures 6 and 7 and Additional files 4 to 17. For instance, cell 1151 carries a known terminal 9.3 Mb duplication at the chromosome 18 p terminus (Figure 6). This duplication is called after channel clone normalization, but not after the other loess-based methods. Figure 7 illustrates that chromosome 21 of cell 1160 is expected to have no aberration. This is confirmed by SNP-array analysis that revealed no loss-of-heterozygosity for this 21q-ter segment. However, the q-terminal region of this chromosome is detected as a deletion after global loess, CGHnormaliter and poplowess normalizations, thus resulting in a false-positive CNV region.

Haarseg is an algorithm integrating signal smoothing, normalization, segmentation, and copy number calling [13]. However, this algorithm performs somewhat conservatively in calling chromosomal aberrations in the single-cell array CGH data, even though it gives a lower FPR than loess-based normalization methods. We also checked the performance of GADA in the single-cell application. GADA is an iterative procedure combining normalization and segmentation by sparse Bayesian learning. Around 800 breakpoints were detected in each EBV-transformed sample by GADA (Additional file 18). This is biologically unrealistic, and we conclude that many false positive aberrations have been detected. Although Haarseg and GADA are suitable in genomic array CGH data [13,15], the implementation of these methods becomes inappropriate for single-cell array CGH data. The channel clone method outperforms these methods, having the largest TPR (0.98) and smallest FPR (0.006). Clearly, channel clone normalization improves the TPR considerably compared to these other normalization algorithms or normalization integrated algorithms for single-cell array CGH.

Recently, a unified model has been developed by the simultaneous integration of normalization, segmentation and copy number calling [16]. This model has been shown to be efficient for genomic array CGH data. The advantage of this model is that it can incorporate existing preprocessing methods into one model. It would be attractive to enrich this model by accounting for single-cell data properties for single-cell CNV detection in the near future.

### Application 2: human embryo array CGH

In reality, the assumption that only few probes display an aneuploidy copy number and most probes display diploid copy numbers does not hold generally (for example, consider heavily rearranged blastomeres, tumor cells, and so on). It is important, therefore, to test whether channel clone normalization would overcorrect the signals of heavily aberrant samples. We applied the channel clone normalization approach to array CGH of14 blastomeres from previously published work [2]. All the blastomeres extracted from human embryo 20 carry multiple aberrations. The confirmed karyotype of each blastomere has been described in the previously published paper.

**Figure 8:**
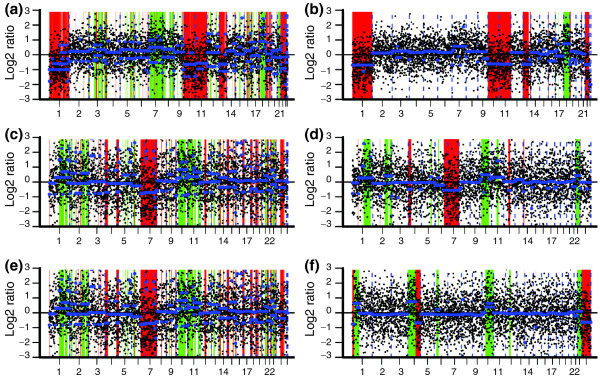
**Copy number variation detection in three blastomere samples**. **(a,c,e) **Genome-wide CNV detection of blastomere A (a), blastomere E (c) and blastomere G (e) from embryo 20 before channel clone normalization. **(b,d,f) **Genome-wide CNV detection of blastomere A (b), blastomere E (d) and blastomere G (f) from embryo 20 after channel clone normalization. The *x*-axis represents the coordinate range from chromosome 1 to × and the *y*-axis represents the log2 ratios. The blue line represents the CBS segmentation line. The green regions represent the duplication region and red regions represent the deletion region called by the CGHcall program.

The results show that many artifacts are observed in the genome profile before channel clone normalization (Figure 8a,c,e). These artifacts were removed after channel clone normalization and none of the real chromosomal aberrations were over-corrected (Figure 8b,d,f). For instance, blastomere A carries aberrations in chromosomes 1, 10, 11, 13, 18, 22, and X, blastomere E carries aberrations in chromosomes 1, 2, 4, 7, 10, 11, and 22, and blastomere G carries aberrations in chromosomes 1, 4, 10, 22 and 23. Figure 8b,d,e shows that all of these aberrations were detected after the channel clone normalization. Thus, channel clone normalization appears valid for heavily aberrant samples as well.

## Discussion

The analysis of CNV in single cells using high-density arrays is a novel attractive research technique [20-23]. It enables genome-wide analysis of blastomeres during early embryogenesis, cell development, and cancer progression [2]. Because the amount of DNA that can be derived from single cells is limited, amplification is necessary. However, amplifying only the test sample results in an amplification bias as well as serious genome artifacts with respect to the log-intensity ratios and leads to poor CNV detection in single-cell array CGH data. So far, no standard procedures have been established to correct this amplification bias and genome artifacts for single cell array CGH. We present a channel clone normalization method that addresses this issue.

The main need for a specific normalization method for single-cell array CGH, as opposed to standard genomic array CGH, arises from the fact that the amplification step in the protocol for single-cell array CGH introduces a key difference compared to array CGH using DNA extracted from a large number of cells. Indeed, only the test sample undergoes DNA amplification while the reference sample remains a DNA sample extracted from a large number of cells with the normal wild-type karyotype. This introduces a major bias in the distribution of signals between the test (amplified single-cell DNA) and reference (non-amplified DNA) samples and genome artifacts, which our method aims to correct. Amplification of the reference sample from a single wild-type cell would be difficult because using amplified single-cell reference samples is unlikely to cancel out the biases caused by amplification since the amplification bias appears to be variable between samples in practice.

Our normalization approach is based on standardization of the distributions of the intensities of test and reference samples, genome composition artifact correction and recurrent genome artifact correction across all the samples. We have shown that our channel clone normalization method clearly improves the performance of single-cell CNV detection compared to other normalization methods, as well as the combined normalization segmentation methods, without losing the ability to detect real aberrations.

## Conclusions

We have proposed a normalization strategy to handle interchannel variation and genome artifacts in two-color arrays and evaluated its applicability using simulated data and data from real single-cell array CGH experiments. Our method was designed originally for single-cell array CGH experiments, but it can be extended to other two-color array experiments that suffer from interchannel variation or genome artifacts. Our approach has the following advantages: first, it achieves good performance for the detection of genomic signals; second, it does not require complex experimental designs, which make the experiments less expensive; and finally, it can be easily implemented without requiring long computing times.

## Materials and methods

### Channel clone normalization

The pre-processing consists of four steps. Step 1, filter clones: the internal control, incorrectly annotated and low foreground-intensity clones are filtered out. Step 2, channel standardization: the log2-transformed intensity of test sample and reference sample are standardized based on the trimmed mean and standardized deviation. Step 3, genome composition artifact correction: log2 ratios are subjected to weighted linear regression on the highest correlated GC content, with larger weights for the GC-rich clones. Step 4, recurrent genome artifact correction: a profile is generated using the trimmed mean of log2 ratios for each probe across all the samples. Subsequently, the common profile trend is estimated by applying a spline model to the generated profile. Finally, the estimated common profile trend is subtracted from each individual genome profile.

The channel clone normalization approach was implemented in R 2.12.1 [24] and the code is available in Additional file 19. The last three steps (channel standardization, genome composition correction and recurrent genome artifact correction) are the core steps of our approach. The impact of each normalization step is discussed in the Results section and the details of each step are explained below.

#### Filtering of clones

First, internal control and clones with incomplete physical annotations are removed. Second, the median background intensities of each array across all the spots are calculated. Subsequently, clones with intensities more than five-fold smaller than the median background intensities as a threshold are filtered out [2]. The threshold is chosen with the help of the MA plot of raw intensities excluding internal control and incomplete physical annotated clones. For instance, Additional file 1 shows the MA plot of the raw intensity of EBV-transformed cell 1160, with the red spots corresponding to clones with intensities more than five-fold smaller than the median background intensity of this array. These low intensity clones show higher variability than the other clones [25] and are thus excluded.

#### Channel standardization

The log2-transformed intensity of the test sample and reference sample are standardized based on the trimmed mean and standard deviation:

Testij_s tandardize=Testij-trimmedmean(Testj)sd(Testj)

Refij_s tandardize=Refij-trimmedmean(Refj)sd(Refj)

where *Test_ij _*represents the log2-transformed intensities of the *i*-th probe of the *j*-th array derived from a test sample; *trimmedmean*(*Test_j_*) represents the trimmed mean of the log2-transformed intensities of the *j*-th array derived from the test sample; *sd*(*Test_j_*) represents the standard deviation of the log2-transformed intensities of the *j*-th array of the test sample; and *Test*_*ij*_*s*tan*dardize *_represents the standardized intensities of the test sample. The parameters to calculate the standardized intensities for the reference sample *Re*f_*ij*_*s*tan*dardize *_are defined in a similar way as for the test sample *Test*_*ij*_*s*tan*dardize *_.

In this step, the amplification bias is expected to be removed by adjusting most of the intensities of the reference and test samples to follow similar distributions without reducing the correlation between them. To make the normalization robust to outliers, the trimmed mean instead of the global mean is calculated. The difference between the mean and trimmed mean is that the mean is calculated using all of the observations whereas the trimmed mean is based on observations excluding a percentage threshold of extreme observations. The trimmed mean is thus less influenced by extreme values than the mean and more robust to outliers. The percentage threshold is determined from a QQ plot between the intensities of the test and reference samples. For instance, the QQ plot in Figure 4 shows that, in our case, approximately 20% of the points have extreme values, located at the two ends of the plot.

#### Genome composition artifact correction

This step aims to correct for genome composition-related artifacts by the weighted linear regression of log2 ratios on the GC content of an enlarged window around probes. The model is stated as follows:

$$Y_{i}=\beta_{0}+\beta_{1}x_{i}+\beta_{2}x_{i}^{2}+\varepsilon_{i}$$

To estimate the parameter *β*, the following expression needs to be minimized:

$$L_{w}\left(\beta \right)=\overset{n}{\underset{i=1}{\sum }}w_{i}\left(Y_{i}-\beta_{0}-\beta_{1}x_{i}-\beta_{2}x_{i}^{2}\right)$$

where *w_i _*is 1,000 if *x_i _*> 0.5 and 0.01 in other cases; *Y_i _*represents the log2 ratio of probe *i *obtained from the 'channel standardization' step; *x_i _*represents the GC content of a certain window size around each probe.

GC contents of different window sizes around probes ranging from 0 to 1 Mb are extracted from the human genome sequence. Next, the correlation between GC content and window size and log2 ratio is calculated. The window size with the highest correlation is selected to fit the model. Thirdly, a large weight (1,000) was assigned to the clones with large GC content whereas a small weight (0.01) was assigned to the clones with low GC content. The residual *ε *of the model is the log2 ratio after the genome composition correction.

#### Recurrent genome artifact correction

This step corrects recurrent genome artifacts. The recurrent genome artifacts are expected to be represented by the estimated common profile across all the samples. Therefore, a common profile is generated by calculating a trimmed mean of log2 ratios for each probe across all the samples. The common profile trend is estimated using a spline smoothing function [26]:

$$S\left(g\right)=\overset{n}{\underset{i=1}{\sum }}{\left\{Y_{i}-g\left(t_{i}\right)\right\}}^{2}+\alpha \int {\left\{g^{\prime \prime }\left(x\right)\right\}}^{2}d\left(x\right)$$

where *i *represents the *i*-th probe of an array; *t_i _*represents the genome physical position of probe *i*;*Y_i _*represents the log2 ratio of probe *i *after genome composition correction; *n *represents the number of knots; *g *represents a twice-differentiable function; *g*(*t_i_*) represents the estimated smoothing value of the log2 ratio from probe *i*; *α *represents a smoothing parameter balancing the model fitting and the model complexity. The maximum of knots that are equal to the total number of probes were used to fit the model.

The smoothing spline estimation of *g*(*t_i_*) is the minimizer of *s*(*g*). It represents the common profile trend across all the samples including recurrent genome artifacts. Thus, the subtraction of the estimated common profile trend from each individual genome profile can remove the recurrent genome artifacts.

### Simulation

A simulation data set was generated based on seven real EBV-transformed samples (described below). Firstly, the EBV data set was processed by setting the true aberrant intensities as empty values. Subsequently, 15 sample intensities were simulated by replacing individual probe intensities from corresponding processed EBV probe intensities. These two steps ensure that all the simulated intensities are non-aberrant. In addition, the simulated genome profiles represent the real single-cell genome profile features, including recurrent genome artifacts. Thirdly, 23 aberrations were artificially added to the simulated data, with the mean intensities of the simulated aberrations setting as the ones of the true aberrant regions from the real EBV-transformed samples. The length of the aberrations was set to around 20 Mb.

### Single EBV-transformed lymphoblastoid cell array CGH

Seven EBV-transformed cells derived from patients carrying known unbalanced chromosomal rearrangements were isolated, lysed and amplified following a multiple displacement amplification approach using Genomi Phi V2 [6]. Amplified single cell and non-amplified genomic DNA (500 ng) derived from a patient with Klinefelter syndrome was labeled for 2 hours by random primer labeling using Cy5 and Cy3 dCTPS and hybridized according to the manufacturer's instructions to the genome-wide Agilent 244 K array. Slides were scanned by Feature Extraction software using Agilent protocol CGH-v4_10_Apr08. As a validation, genomic DNA isolated from multiple cells of the corresponding EBV-transformed lines was karyotyped as well as analyzed on a 250 K Affymetrix SNP array to confirm real aberrant regions. A deleted region on a SNP array presents only a single allele and is indicated by loss-of-heterozygosity. Diploid regions were confirmed by heterozygous SNPs [2]. The karyotype of each EBV-transformed sample is listed in Table 1.

### Human embryo array CGH

Fourteen blastomeres derived from human embryo 6, 8, 15, 16, 19 and 20 carrying known chromosomal rearrangements were hybridized to the Agilent 244 K array. The experimental protocol and validation were similar to the single EBV-transformed cell array CGH and are explained in detail in previously published work [2]. Most of these blastomeres carry multiple aberrations within one cell. The complete karyotype of each blastomere is reported in [2].

### Gene Expression Omnibus accession numbers

All the single-cell data from this study are public accessible in the Gene Expression Omnibus under SuperSeries [GSE31219]. GSE31219 contains single EBV-transformed lymphoblastoid cell array and human blastomere array data. The previously published human blastomere data are accessible through Gene Expression Omnibus series accession number [GSE11663].

### Evaluation of CNV calling in single-cell array CGH experiments

The parameters of the CBS algorithm were optimized to detect validated known CNVs of EBV-transformed single cells. The CGHcall program was used to call the CNVs in single cells. It fits each CBS segment to a mixture model with four states and calls each segment as a duplication, deletion, amplification or normal state [27]. We calculated the TPR and FPR to evaluate the CNV detection. TPR was defined as the length of CGHcall CNVs within the true aberrant regions divided by the total length of true aberrant regions. FPR was defined as the length of CGHcall CNVs outside the aberrant regions divided by the total non-aberrant region lengths [28]. The CBS algorithm was implemented using the R package snapCGH [9].

## Abbreviations

CBS: circular binary segmentation; CGH: comparative genomic hybridization; CNV: copy number variation; EBV: Epstein-Barr virus; FPR: false positive rate; GADA: genome alteration detection analysis; QQ: quantile-quantile; SD: standard deviation; SNP: single nucleotide polymorphism; TPR: true positive rate.

## Authors' contributions

JRV designed the novel single-cell experiment and critically reviewed the manuscript. EV and TV designed the novel single-cell experiment, carried out the single-cell experiments and generated the array data and critically reviewed the manuscript. PK was involved in the statistical discussion and critically reviewed the manuscript. YM conceived of the study, guided JC to develop the algorithm and critically reviewed the manuscript. JC performed the analysis and wrote the manuscript. All authors have read and approved the final manuscript.

## Supplementary Material

Additional file 1 **Figure S1 - MA plot of single-cell array CGH**. MA plot of EBV-transformed cell 1160. The spots in the plot are the clones excluding internal control and incomplete physical annotated clones. The red spots represent clones with intensities more than five-fold lower than the median background intensity. Click here for file

Additional file 2 **Figure S2 - genome profile of single-cell array CGH before and after genome composition correction**. Genome plots of EBV-transformed cell 1168. **(a,b) **Genome plots of chromosomes 1 and 10 before genome composition correction. **(c,d) **Genome plots of chromosomes 1 and 10 after genome composition correction. The red line represents a lowess curve fitted to the data. Click here for file

Additional file 3 **Figure S3 - genome profile of single-cell array CGH before and after recurrent genome artifacts correction**. **(a) **Genome plot of chromosome 3 from EBV-transformed cell 1168 before recurrent genome artifact correction. The red line represents the CBS segmentation. **(b) **Estimated common profile trend of chromosome 3 across all the EBV-transformed cells. The red line represents a lowess curve. **(c) **Genome plot of chromosome 3 from EBV-transformed cell 1168 after recurrent genome artifact correction. The red line represents the CBS segmentation. Click here for file

Additional file 4 **Figure S4 - genome-wide copy number variation detection of single EBV-transformed cell 1168 using existing normalization methods**. **(a-d) **Single-cell CNV detection of EBV-transformed cell 1168 after global loess (a), CGHnormaliter (b), poplowess (c) and Haarseg normalization (d). The *y*-axis represents the log2 ratios and the *x*-axis the probe position along the chromosome. The blue line represents the CBS segmentation line. The red region represents the deletion and the green region represents the duplication called by CGHcall. Click here for file

Additional file 5 **Figure S5 - genome-wide copy number variation detection of single EBV-transformed cell 1151 using existing normalization methods**. **(a-d) **Single-cell CNV detection of EBV-transformed cell 1151 after global loess (a), CGHnormaliter (b), poplowess (c) and Haarseg normalization (d). The *y*-axis represents the log2 ratios and the *x*-axis the probe position along the chromosome. The blue line represents the CBS segmentation line. The red region represents the deletion and the green region represents the duplication called by CGHcall. Click here for file

Additional file 6 **Figure S6 - genome-wide copy number variation detection of single EBV-transformed cell 1160 using existing normalization methods**. **(a-d) **Single-cell CNV detection of EBV-transformed cell 1160 after global loess (a), CGHnormaliter (b), poplowess (c) and Haarseg normalization (d). The *y*-axis represents the log2 ratios and the *x*-axis the probe position along the chromosome. The blue line represents the CBS segmentation line. The red region represents the deletion and the green region represents the duplication called by CGHcall. Click here for file

Additional file 7 **Figure S7 - genome-wide copy number variation detection of single EBV-transformed cell 1162 using existing normalization methods**. **(a-d) **Single-cell CNV detection of EBV-transformed cell 1162 after global loess (a), CGHnormaliter (b), poplowess (c) and Haarseg normalization (d). The *y*-axis represents the log2 ratios and the *x*-axis the probe position along the chromosome. The blue line represents the CBS segmentation line. The red region represents the deletion and the green region represents the duplication called by CGHcall. Click here for file

Additional file 8 **Figure S8 - genome-wide copy number variation detection of single EBV-transformed cell 614 using existing normalization methods**. **(a-d) **Single-cell CNV detection of EBV-transformed cell 614 after global loess (a), CGHnormaliter (b), poplowess (c) and Haarseg normalization (d). The *y*-axis represents the log2 ratios and the *x*-axis the probe position along the chromosome. The blue line represents the CBS segmentation line. The red region represents the deletion and the green region represents the duplication called by CGHcall. Click here for file

Additional file 9 **Figure S9 - genome-wide copy number variation detection of single EBV-transformed cell 617 using existing normalization methods**. **(a-d) **Single-cell CNV detection of EBV-transformed cell 617 after global loess (a), CGHnormaliter (b), poplowess (c) and Haarseg normalization (d). The *y*-axis represents the log2 ratios and the *x*-axis the probe position along the chromosome. The blue line represents the CBS segmentation line. The red region represents the deletion and the green region represents the duplication called by CGHcall. Click here for file

Additional file 10 **Figure S10 - genome-wide copy number variation detection of single EBV-transformed cell 1013 using existing normalization methods**. **(a-d) **Single-cell CNV detection of EBV-transformed cell 1013 after global loess (a), CGHnormaliter (b), poplowess (c) and Haarseg normalization (d). The *y*-axis represents the log2 ratios and the *x*-axis the probe position along the chromosome. The blue line represents the CBS segmentation line. The red region represents the deletion and the green region represents the duplication called by CGHcall. Click here for file

Additional file 11 **Figures S11 - genome-wide copy number variation detection of single EBV-transformed cell 1168 using the channel clone normalization method**. Single-cell CNV detection of EBV-transformed cell 1168 after channel clone normalization. The *y*-axis represents the log2 ratios and the *x*-axis the probe position along the chromosome. The blue line represents the CBS segmentation line. The red region represents the deletion and the green region represents the duplication called by CGHcall. Click here for file

Additional file 12 **Figures S12 - genome-wide copy number variation detection of single EBV-transformed cell 1151 using the channel clone normalization method**. Single-cell CNV detection of EBV-transformed cell 1151 after channel clone normalization. The *y*-axis represents the log2 ratios and the *x*-axis the probe position along the chromosome. The blue line represents the CBS segmentation line. The red region represents the deletion and the green region represents the duplication called by CGHcall. Click here for file

Additional file 13 **Figures S13 - genome-wide copy number variation detection of single EBV-transformed cell 1160 using the channel clone normalization method**. Single-cell CNV detection of EBV-transformed cell 1160 after channel clone normalization. The *y*-axis represents the log2 ratios and the *x*-axis the probe position along the chromosome. The blue line represents the CBS segmentation line. The red region represents the deletion and the green region represents the duplication called by CGHcall. Click here for file

Additional file 14 **Figures S14 - genome-wide copy number variation detection of single EBV-transformed cell 1162 using the channel clone normalization method**. Single-cell CNV detection of EBV-transformed cell 1162 after channel clone normalization. The *y*-axis represents the log2 ratios and the *x*-axis the probe position along the chromosome. The blue line represents the CBS segmentation line. The red region represents the deletion and the green region represents the duplication called by CGHcall. Click here for file

Additional file 15 **Figures S15 - genome-wide copy number variation detection of single EBV-transformed cell 614 using the channel clone normalization method**. Single-cell CNV detection of EBV-transformed cell 614 after channel clone normalization. The *y*-axis represents the log2 ratios and the *x*-axis the probe position along the chromosome. The blue line represents the CBS segmentation line. The red region represents the deletion and the green region represents the duplication called by CGHcall. Click here for file

Additional file 16 **Figures S16 - genome-wide copy number variation detection of single EBV-transformed cell 617 using the channel clone normalization method**. Single-cell CNV detection of EBV-transformed cell 617 after channel clone normalization. The *y*-axis represents the log2 ratios and the *x*-axis the probe position along the chromosome. The blue line represents the CBS segmentation line. The red region represents the deletion and the green region represents the duplication called by CGHcall. Click here for file

Additional file 17 **Figures S17 - genome-wide copy number variation detection of single EBV-transformed cell 1013 using the channel clone normalization method**. Single-cell CNV detection of EBV-transformed cell 1013 after channel clone normalization. The *y*-axis represents the log2 ratios and the *x*-axis the probe position along the chromosome. The blue line represents the CBS segmentation line. The red region represents the deletion and the green region represents the duplication called by CGHcall. Click here for file

Additional file 18 **Figure S18 - genome-wide copy number variation detection of single EBV-transformed cells using the GADA algorithm**. Single-cell CNV detection of all seven EBV-transformed cells. Each row represents the profile of one EBV-transformed cell and each column represents one probe across all the EBV-transformed samples. Different colors in the profile represent the breakpoints of single-cell CNVs detected by GADA. Click here for file

Additional file 19 **R code to implement channel clone normalization approach**. Click here for file
