# Supplementary figures and images for: Single-cell copy number variation detection
Source: Genome Biol. 2011 Aug 19;12(8):R80. doi: 10.1186/gb-2011-12-8-r80 (PMC3245619; doi:10.1186/gb-2011-12-8-r80)

Sup Figure 1

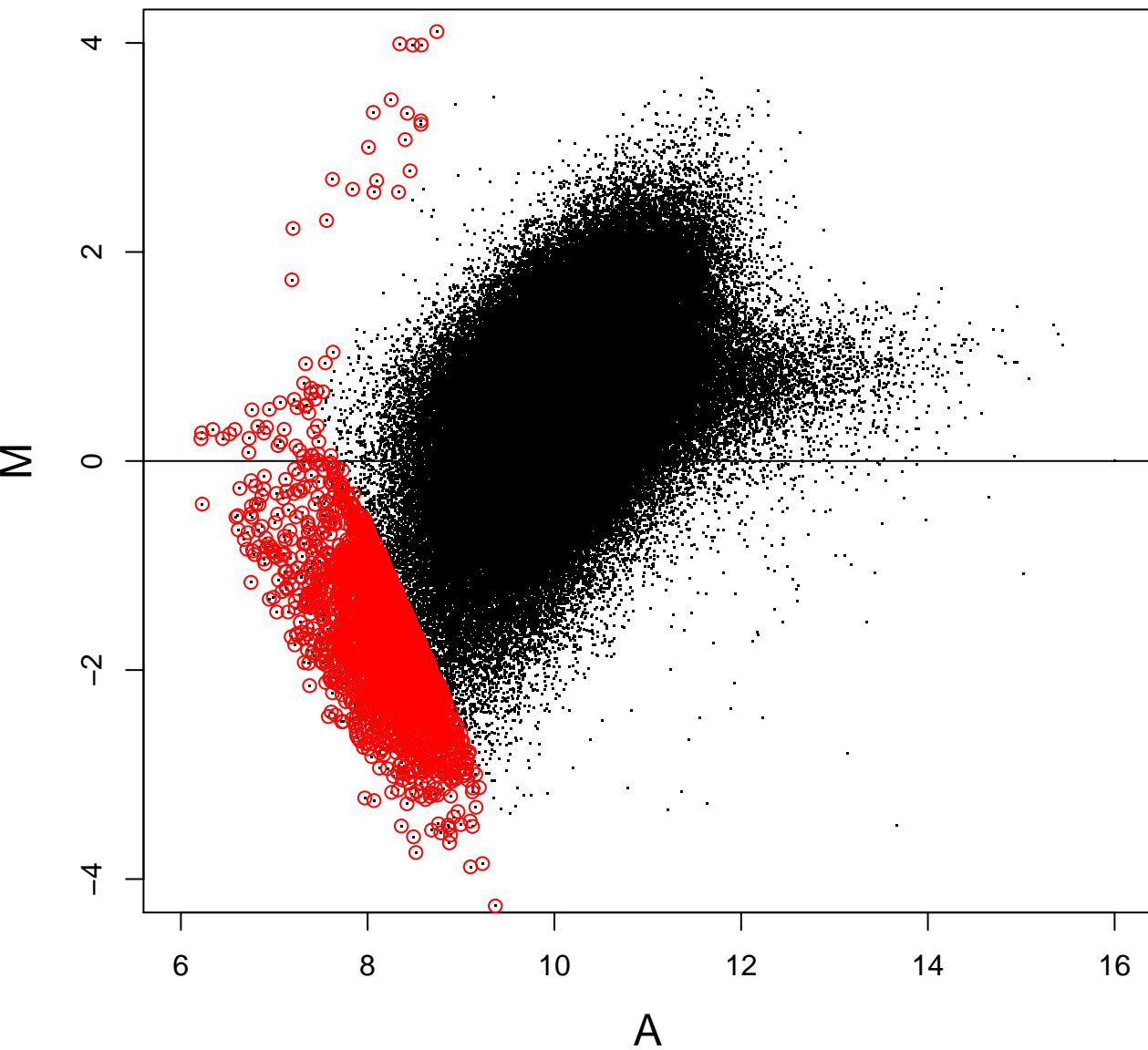

Supplement: Additional file 1 — Figure S1 - MA plot of single-cell array CGH. MA plot of EBV-transformed cell 1160. The spots in the plot are the clones excluding internal control and incomplete physical annotated clones. The red spots represent clones with intensities more than five-fold lower than the median background intensity. [file gb-2011-12-8-r80-S1.PDF]

Sup Figure 2

(A)

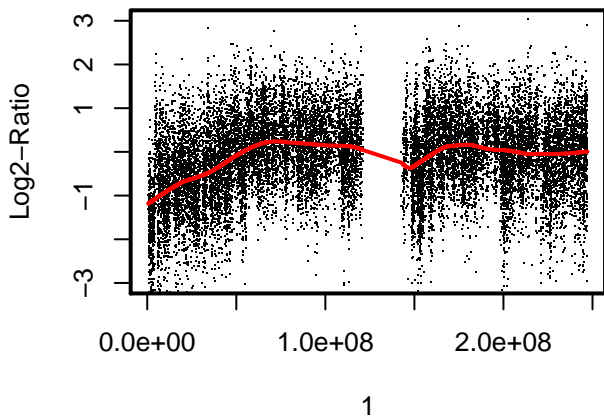

(B)

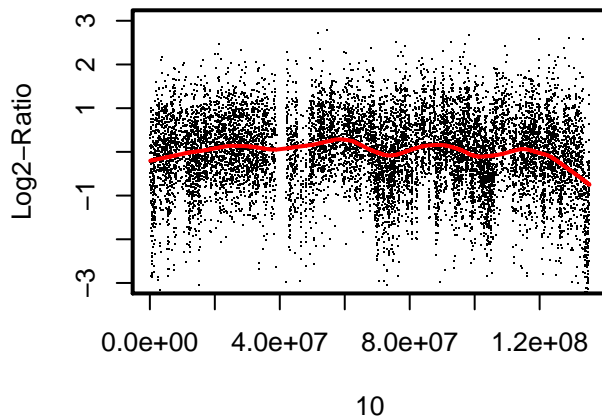

(C)

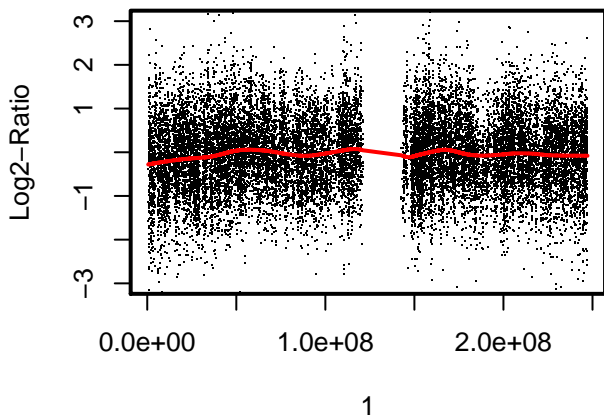

(D)

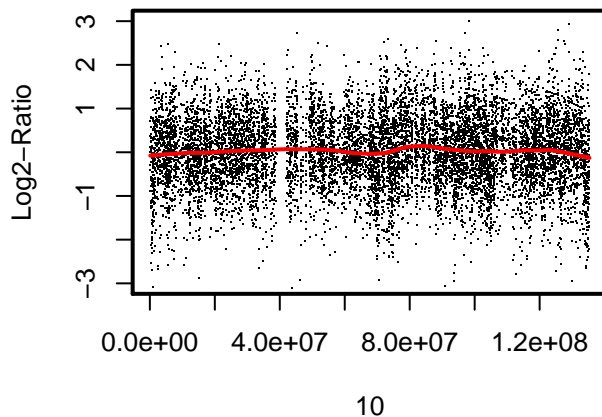

Supplement: Additional file 2 — Figure S2 - genome profile of single-cell array CGH before and after genome composition correction. Genome plots of EBV-transformed cell 1168. (a,b) Genome plots of chromosomes 1 and 10 before genome composition correction. (c,d) Genome plots of chromosomes 1 and 10 after genome composition correction. The red line represents a lowess curve fitted to the data. [file gb-2011-12-8-r80-S2.PDF]

Sup Figure 3

(A)

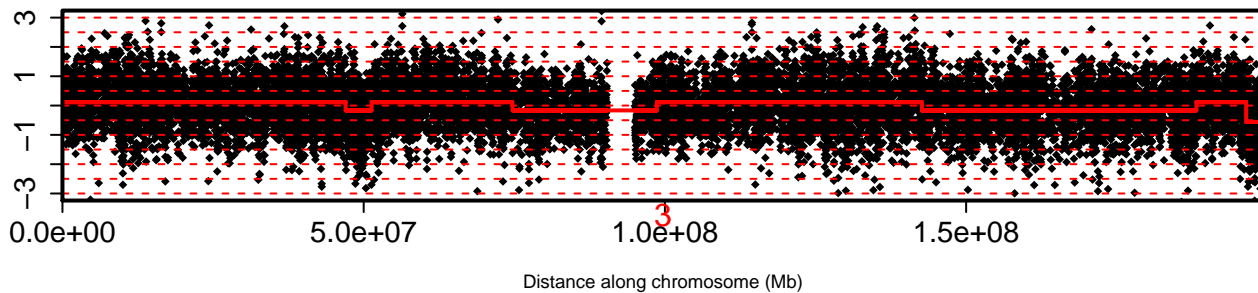

(B)

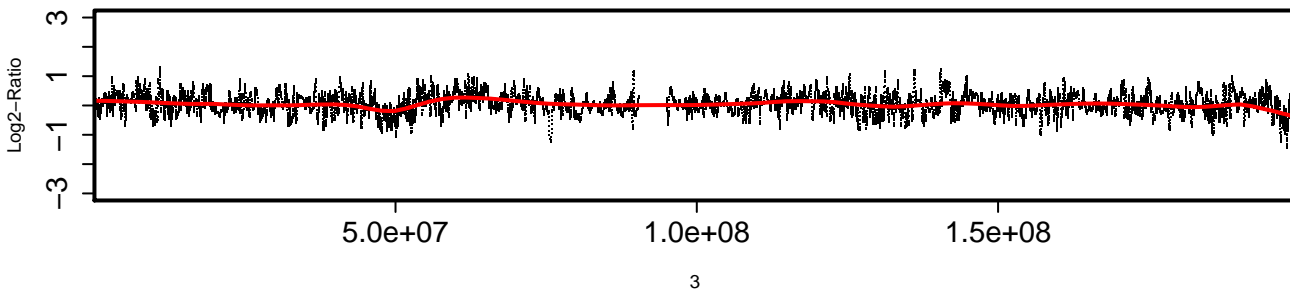

(C)

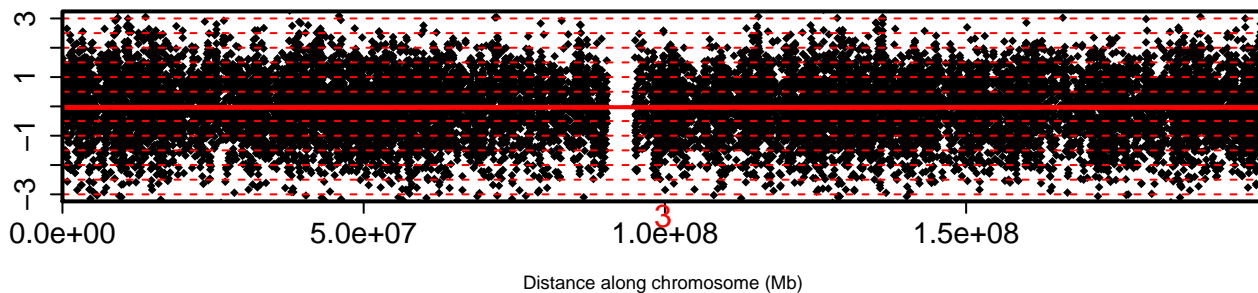

Supplement: Additional file 3 — Figure S3 - genome profile of single-cell array CGH before and after recurrent genome artifacts correction. (a) Genome plot of chromosome 3 from EBV-transformed cell 1168 before recurrent genome artifact correction. The red line represents the CBS segmentation. (b) Estimated common profile trend of chromosome 3 across all the EBV-transformed cells. The red line represents a lowess curve. (c) Genome plot of chromosome 3 from EBV-transformed cell 1168 after recurrent genome artifact correction. The red line represents the CBS segmentation. [file gb-2011-12-8-r80-S3.PDF]

Sup Figure 4

(A)

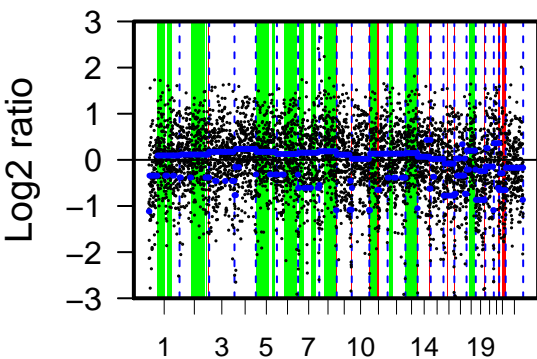

(B)

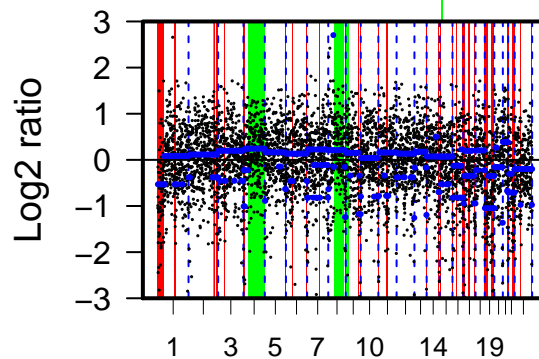

(C)

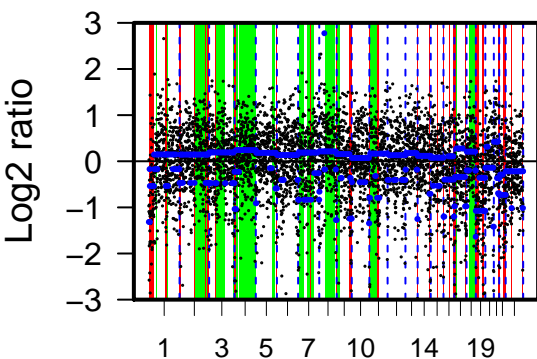

(D)

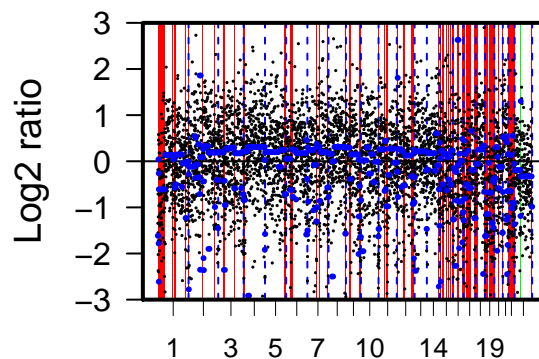

Supplement: Additional file 4 — Figure S4 - genome-wide copy number variation detection of single EBV-transformed cell 1168 using existing normalization methods. (a-d) Single-cell CNV detection of EBV-transformed cell 1168 after global loess (a), CGHnormaliter (b), poplowess (c) and Haarseg normalization (d). The y-axis represents the log2 ratios and the x-axis the probe position along the chromosome. The blue line represents the CBS segmentation line. The red region represents the deletion and the green region represents the duplication called by CGHcall. [file gb-2011-12-8-r80-S4.PDF]

Sup Figure 5

(A)

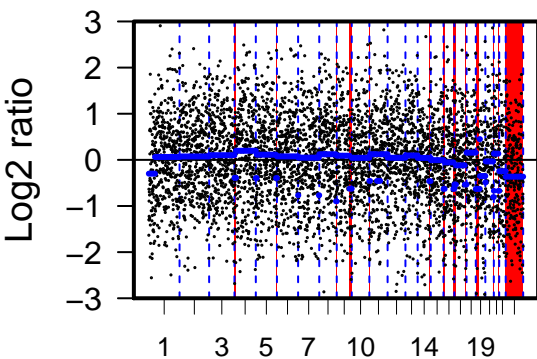

(B)

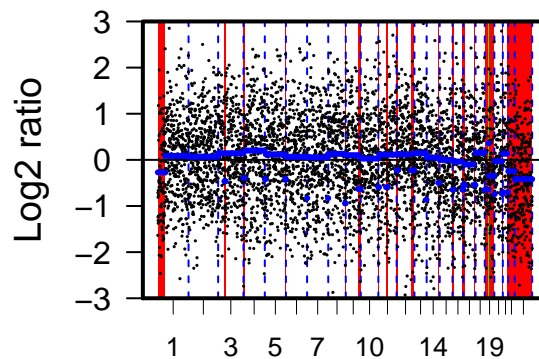

(C)

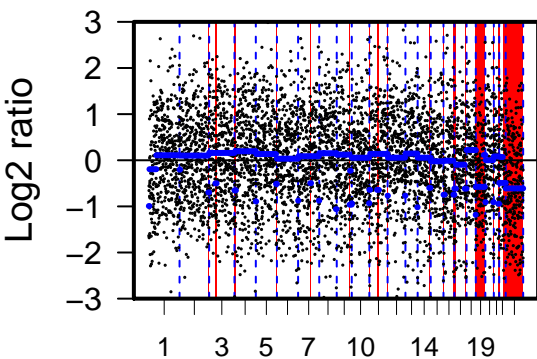

(D)

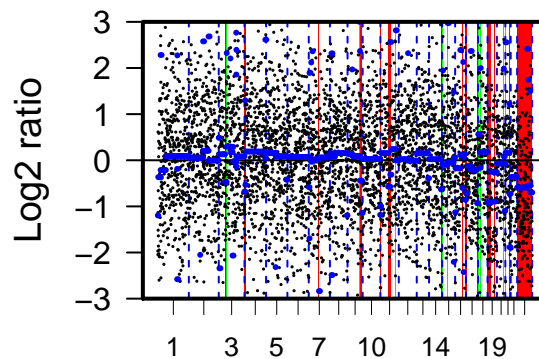

Supplement: Additional file 5 — Figure S5 - genome-wide copy number variation detection of single EBV-transformed cell 1151 using existing normalization methods. (a-d) Single-cell CNV detection of EBV-transformed cell 1151 after global loess (a), CGHnormaliter (b), poplowess (c) and Haarseg normalization (d). The y-axis represents the log2 ratios and the x-axis the probe position along the chromosome. The blue line represents the CBS segmentation line. The red region represents the deletion and the green region represents the duplication called by CGHcall. [file gb-2011-12-8-r80-S5.PDF]

Sup Figure 6

(A)

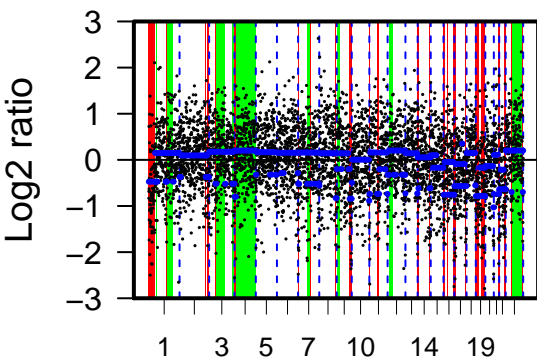

(B)

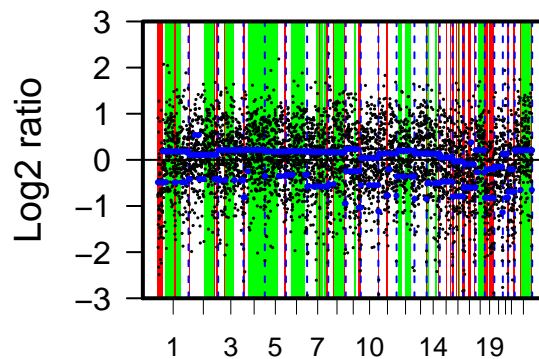

(C)

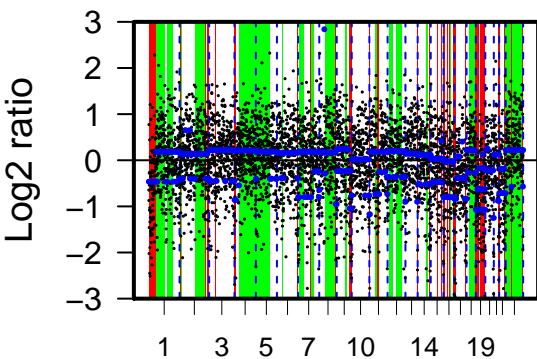

(D)

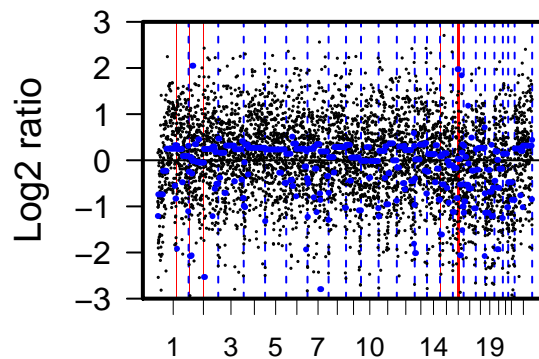

Supplement: Additional file 6 — Figure S6 - genome-wide copy number variation detection of single EBV-transformed cell 1160 using existing normalization methods. (a-d) Single-cell CNV detection of EBV-transformed cell 1160 after global loess (a), CGHnormaliter (b), poplowess (c) and Haarseg normalization (d). The y-axis represents the log2 ratios and the x-axis the probe position along the chromosome. The blue line represents the CBS segmentation line. The red region represents the deletion and the green region represents the duplication called by CGHcall. [file gb-2011-12-8-r80-S6.PDF]

Sup Figure 7

(A)

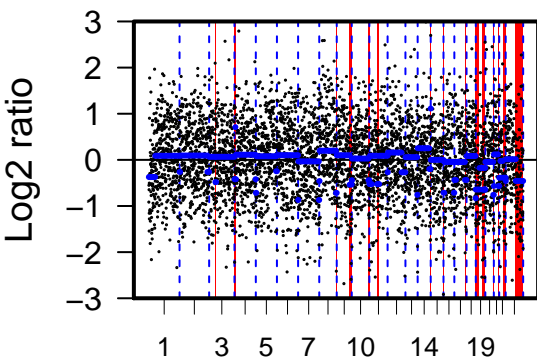

(B)

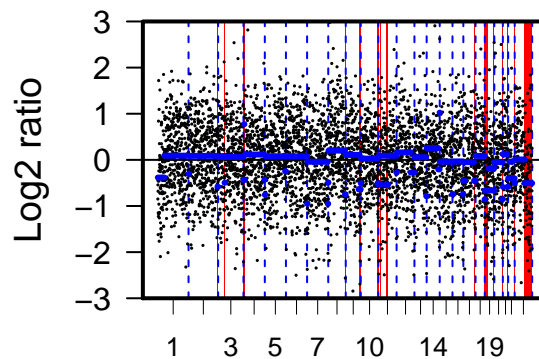

(C)

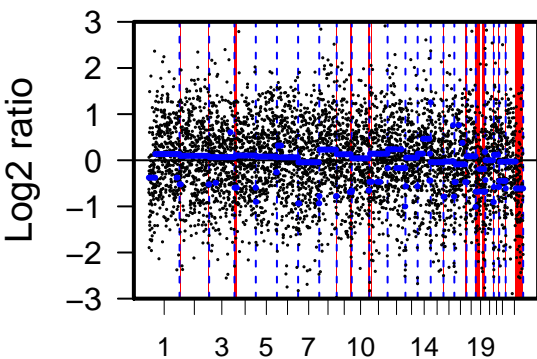

(D)

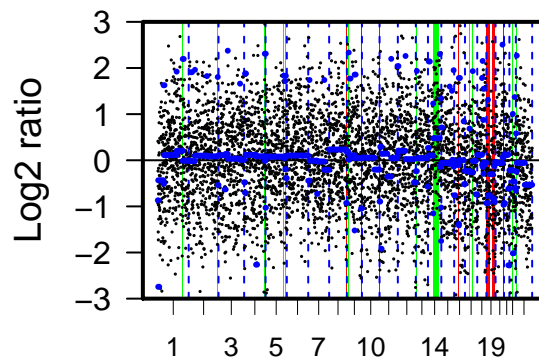

Supplement: Additional file 7 — Figure S7 - genome-wide copy number variation detection of single EBV-transformed cell 1162 using existing normalization methods. (a-d) Single-cell CNV detection of EBV-transformed cell 1162 after global loess (a), CGHnormaliter (b), poplowess (c) and Haarseg normalization (d). The y-axis represents the log2 ratios and the x-axis the probe position along the chromosome. The blue line represents the CBS segmentation line. The red region represents the deletion and the green region represents the duplication called by CGHcall. [file gb-2011-12-8-r80-S7.PDF]

Sup Figure 8

(A)

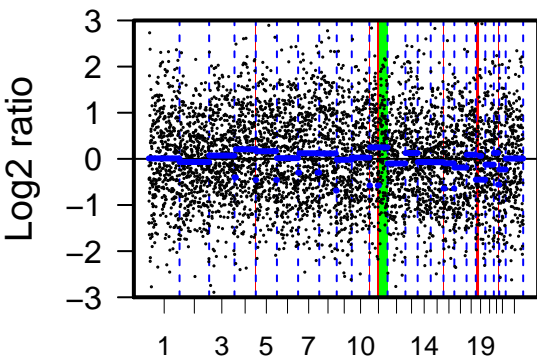

(B)

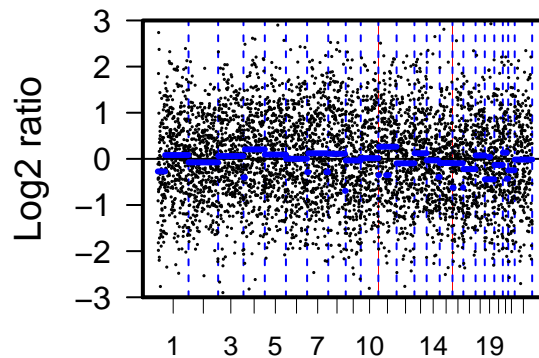

(C)

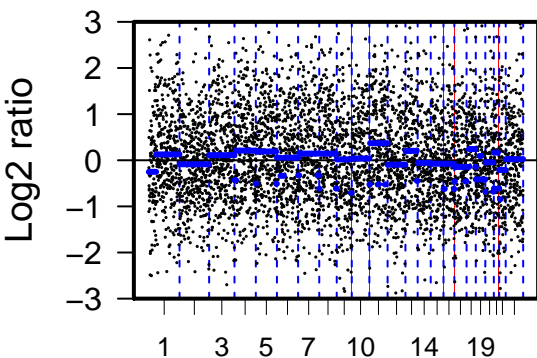

(D)

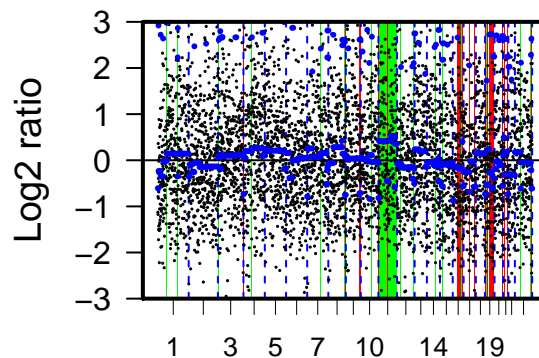

Supplement: Additional file 8 — Figure S8 - genome-wide copy number variation detection of single EBV-transformed cell 614 using existing normalization methods. (a-d) Single-cell CNV detection of EBV-transformed cell 614 after global loess (a), CGHnormaliter (b), poplowess (c) and Haarseg normalization (d). The y-axis represents the log2 ratios and the x-axis the probe position along the chromosome. The blue line represents the CBS segmentation line. The red region represents the deletion and the green region represents the duplication called by CGHcall. [file gb-2011-12-8-r80-S8.PDF]

Sup Figure 9

(A)

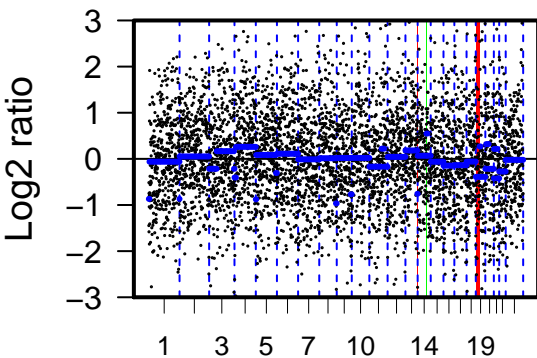

(B)

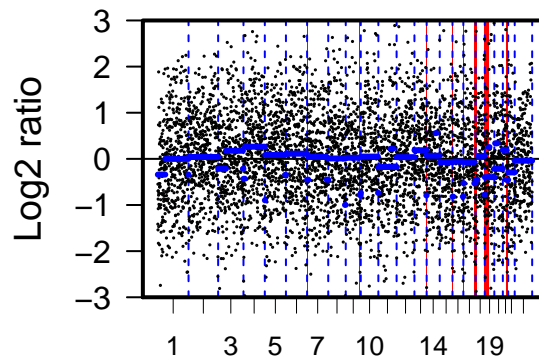

(C)

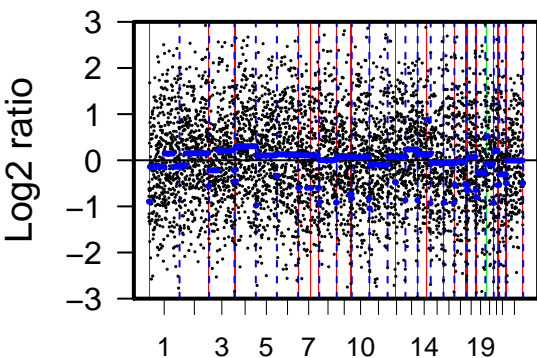

(D)

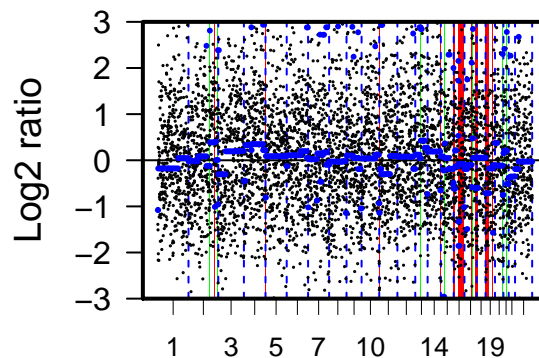

Supplement: Additional file 9 — Figure S9 - genome-wide copy number variation detection of single EBV-transformed cell 617 using existing normalization methods. (a-d) Single-cell CNV detection of EBV-transformed cell 617 after global loess (a), CGHnormaliter (b), poplowess (c) and Haarseg normalization (d). The y-axis represents the log2 ratios and the x-axis the probe position along the chromosome. The blue line represents the CBS segmentation line. The red region represents the deletion and the green region represents the duplication called by CGHcall. [file gb-2011-12-8-r80-S9.PDF]

Sup Figure 10

(A)

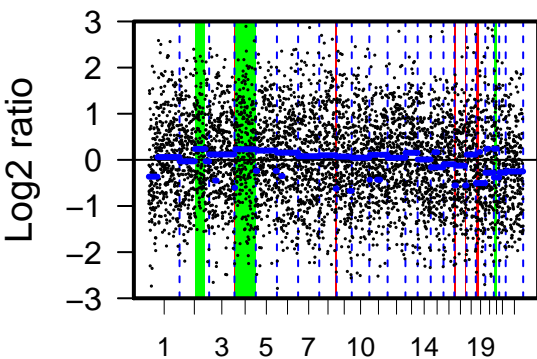

(B)

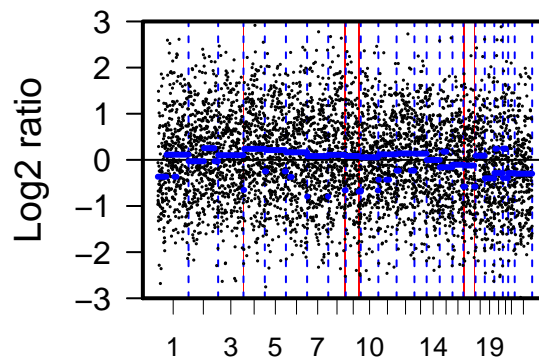

(C)

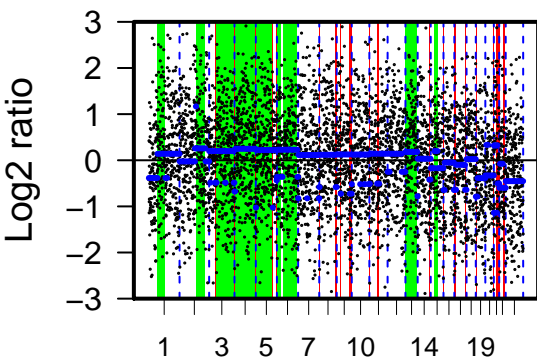

(D)

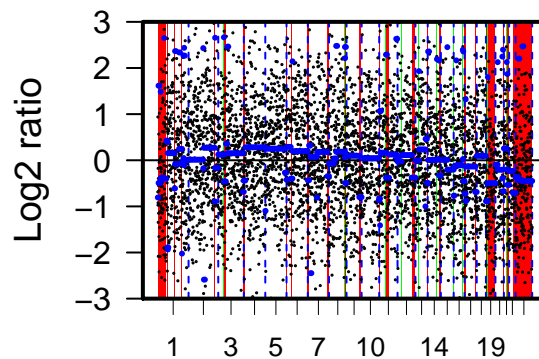

Supplement: Additional file 10 — Figure S10 - genome-wide copy number variation detection of single EBV-transformed cell 1013 using existing normalization methods. (a-d) Single-cell CNV detection of EBV-transformed cell 1013 after global loess (a), CGHnormaliter (b), poplowess (c) and Haarseg normalization (d). The y-axis represents the log2 ratios and the x-axis the probe position along the chromosome. The blue line represents the CBS segmentation line. The red region represents the deletion and the green region represents the duplication called by CGHcall. [file gb-2011-12-8-r80-S10.PDF]

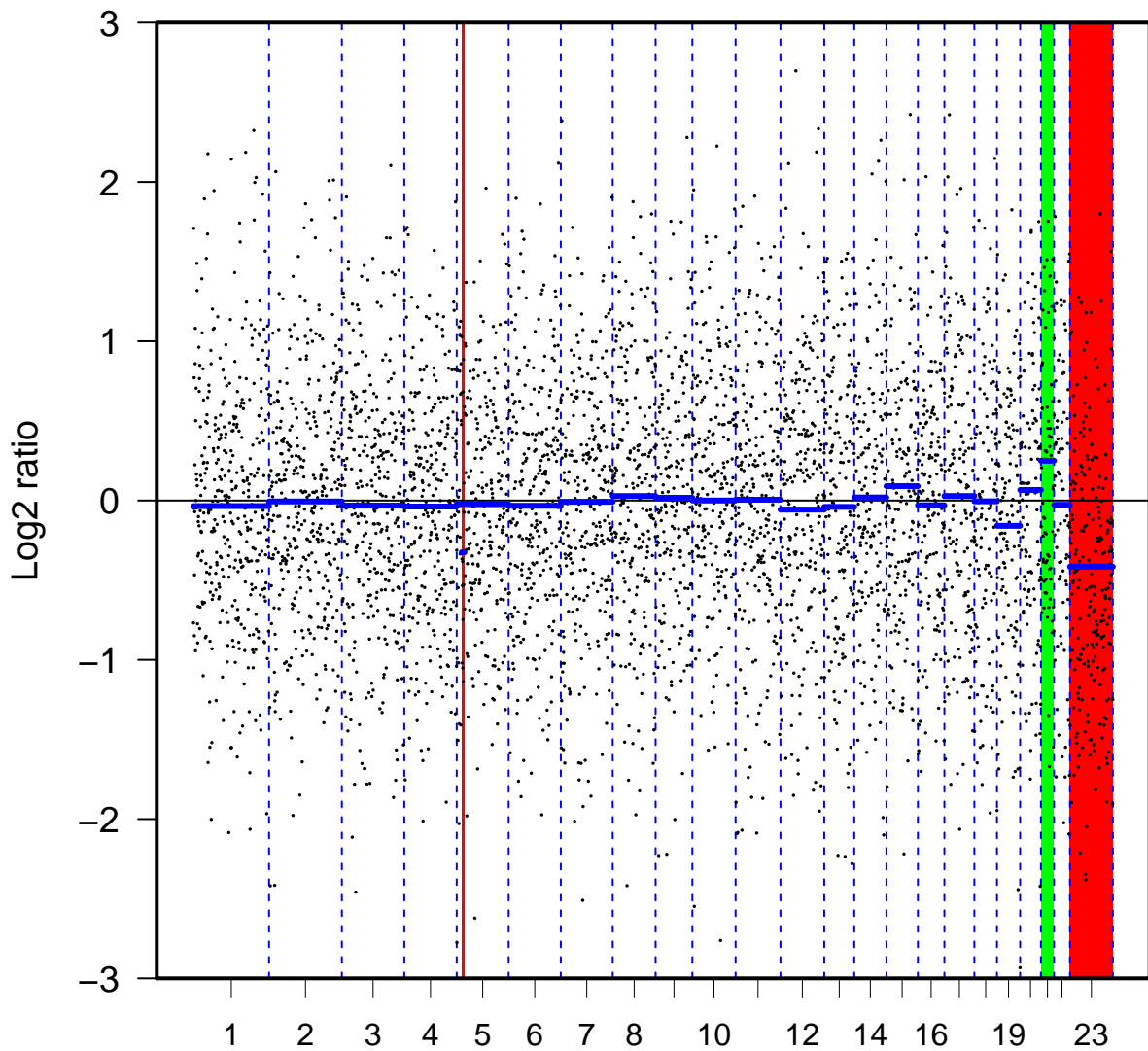

Supplement: Additional file 11 — Figures S11 - genome-wide copy number variation detection of single EBV-transformed cell 1168 using the channel clone normalization method. Single-cell CNV detection of EBV-transformed cell 1168 after channel clone normalization. The y-axis represents the log2 ratios and the x-axis the probe position along the chromosome. The blue line represents the CBS segmentation line. The red region represents the deletion and the green region represents the duplication called by CGHcall. [file gb-2011-12-8-r80-S11.PDF]

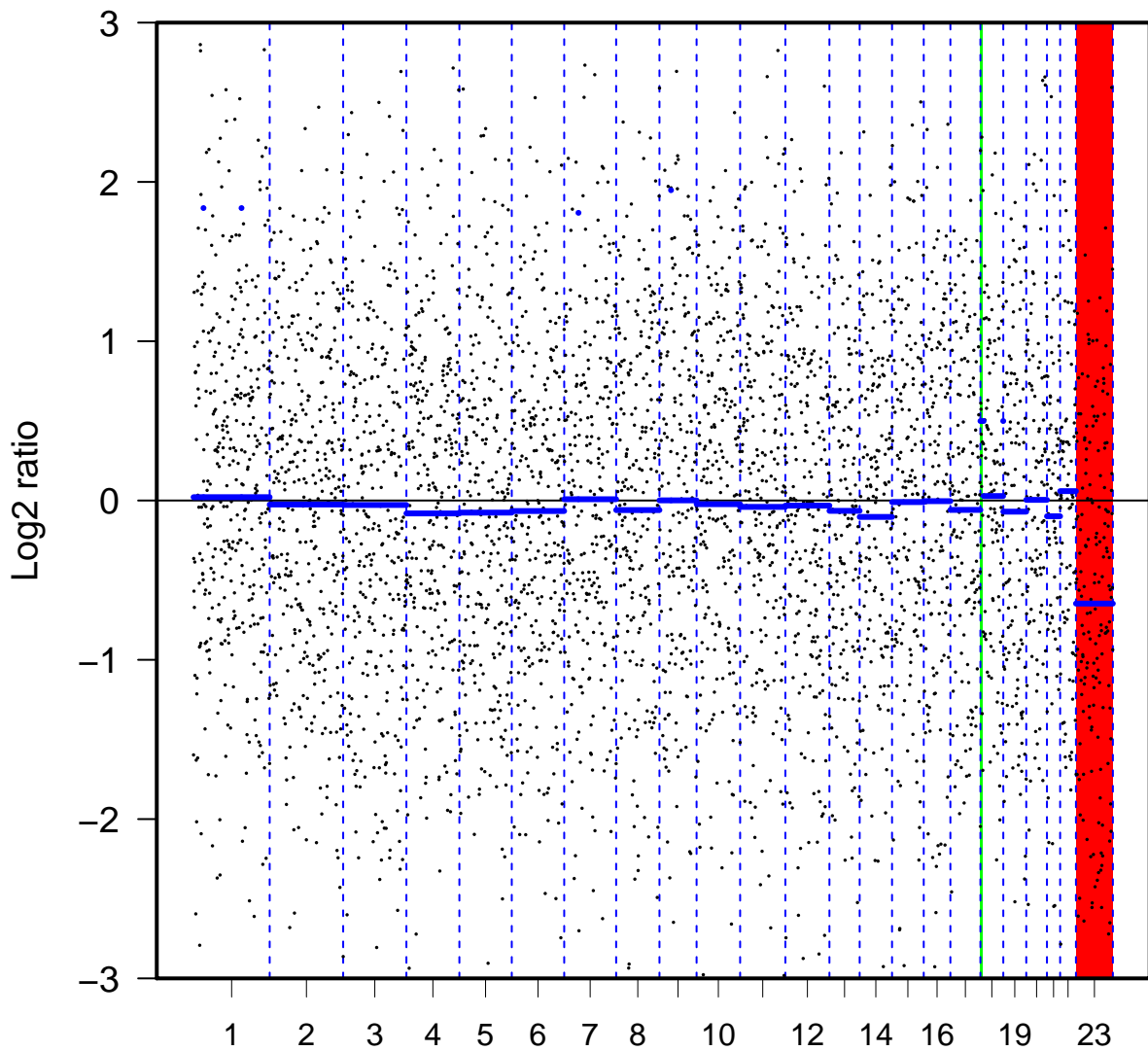

Supplement: Additional file 12 — Figures S12 - genome-wide copy number variation detection of single EBV-transformed cell 1151 using the channel clone normalization method. Single-cell CNV detection of EBV-transformed cell 1151 after channel clone normalization. The y-axis represents the log2 ratios and the x-axis the probe position along the chromosome. The blue line represents the CBS segmentation line. The red region represents the deletion and the green region represents the duplication called by CGHcall. [file gb-2011-12-8-r80-S12.PDF]

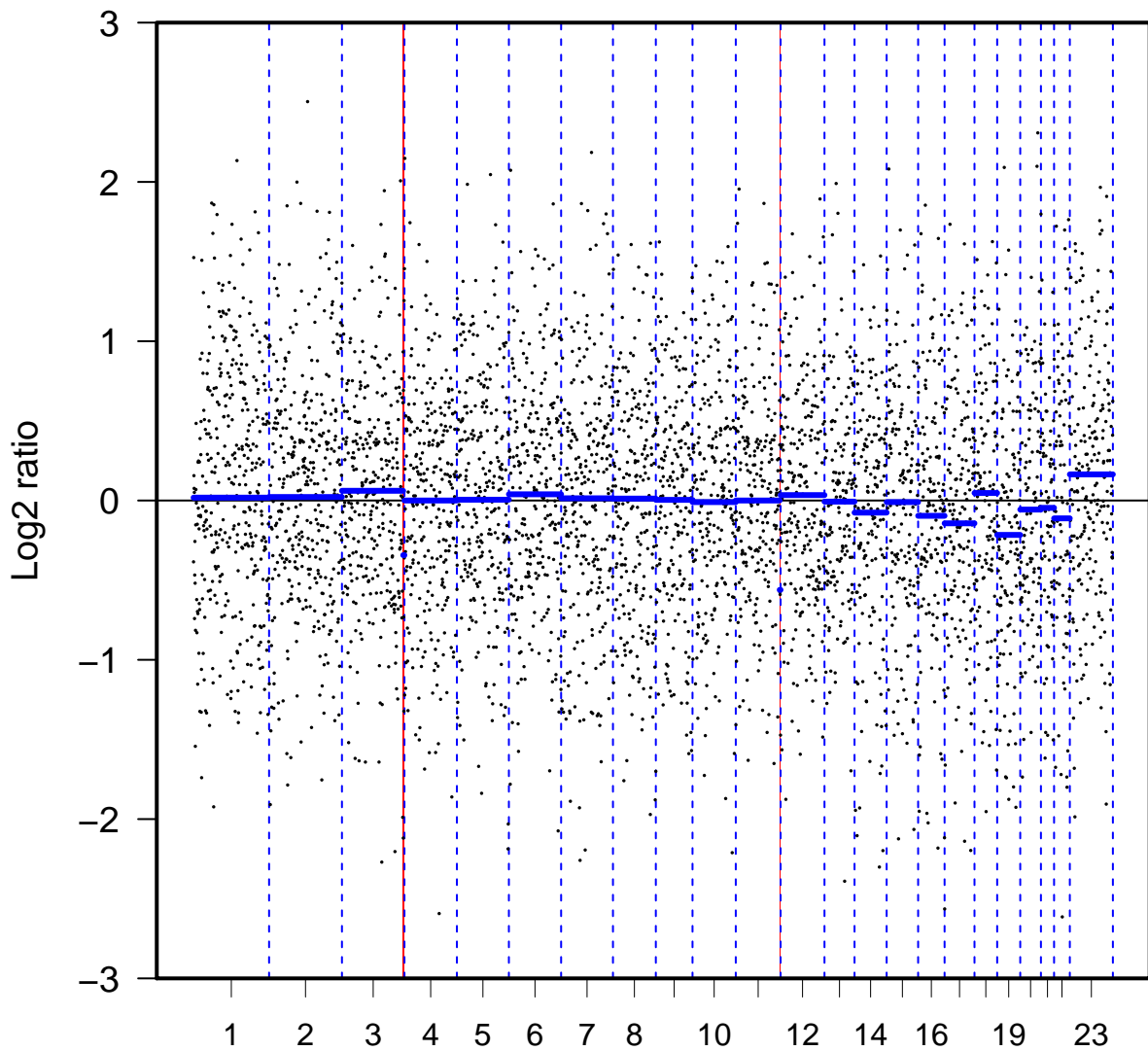

Supplement: Additional file 13 — Figures S13 - genome-wide copy number variation detection of single EBV-transformed cell 1160 using the channel clone normalization method. Single-cell CNV detection of EBV-transformed cell 1160 after channel clone normalization. The y-axis represents the log2 ratios and the x-axis the probe position along the chromosome. The blue line represents the CBS segmentation line. The red region represents the deletion and the green region represents the duplication called by CGHcall. [file gb-2011-12-8-r80-S13.PDF]

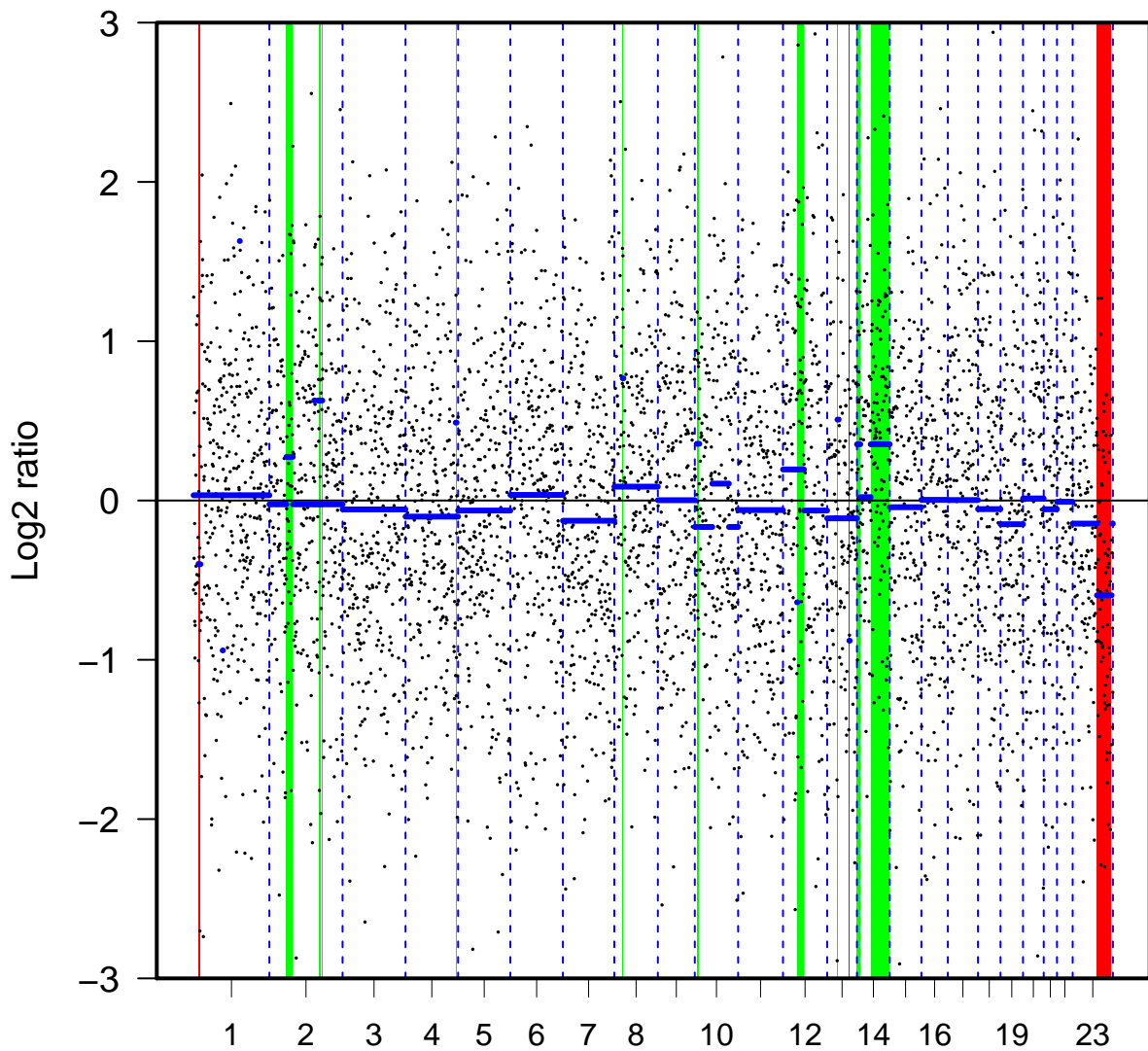

Supplement: Additional file 14 — Figures S14 - genome-wide copy number variation detection of single EBV-transformed cell 1162 using the channel clone normalization method. Single-cell CNV detection of EBV-transformed cell 1162 after channel clone normalization. The y-axis represents the log2 ratios and the x-axis the probe position along the chromosome. The blue line represents the CBS segmentation line. The red region represents the deletion and the green region represents the duplication called by CGHcall. [file gb-2011-12-8-r80-S14.PDF]

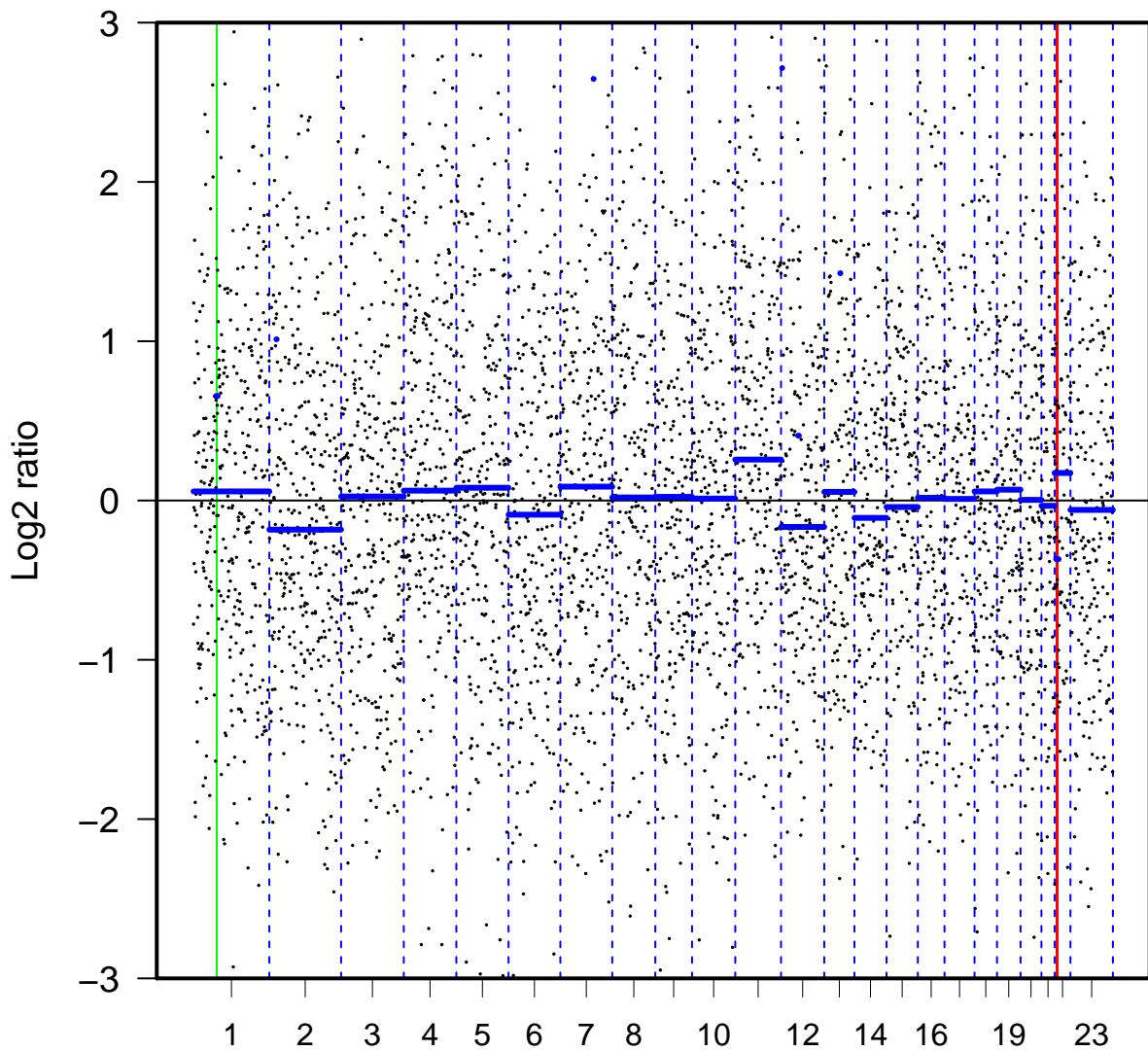

Supplement: Additional file 15 — Figures S15 - genome-wide copy number variation detection of single EBV-transformed cell 614 using the channel clone normalization method. Single-cell CNV detection of EBV-transformed cell 614 after channel clone normalization. The y-axis represents the log2 ratios and the x-axis the probe position along the chromosome. The blue line represents the CBS segmentation line. The red region represents the deletion and the green region represents the duplication called by CGHcall. [file gb-2011-12-8-r80-S15.PDF]

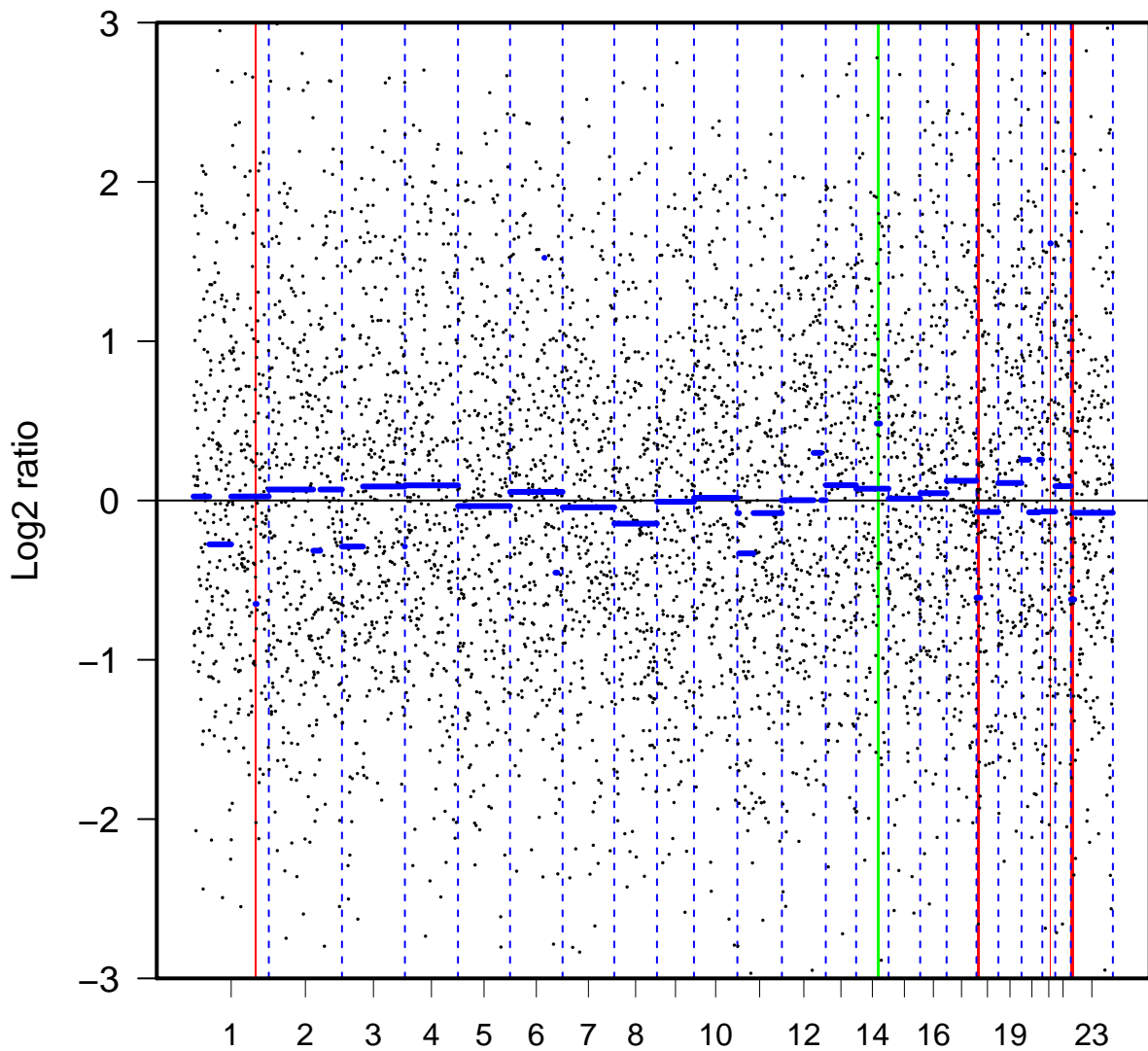

Supplement: Additional file 16 — Figures S16 - genome-wide copy number variation detection of single EBV-transformed cell 617 using the channel clone normalization method. Single-cell CNV detection of EBV-transformed cell 617 after channel clone normalization. The y-axis represents the log2 ratios and the x-axis the probe position along the chromosome. The blue line represents the CBS segmentation line. The red region represents the deletion and the green region represents the duplication called by CGHcall. [file gb-2011-12-8-r80-S16.PDF]

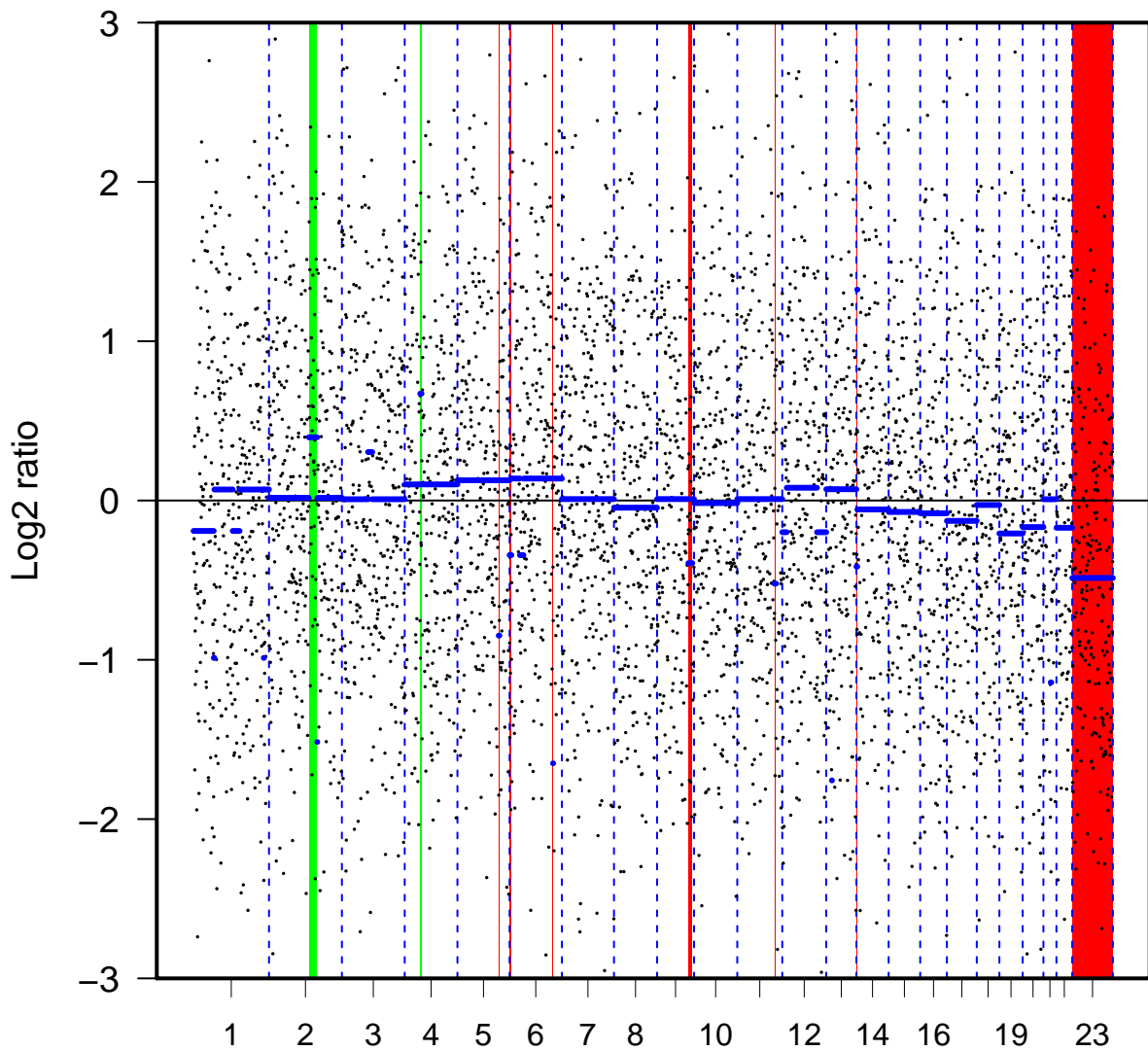

Supplement: Additional file 17 — Figures S17 - genome-wide copy number variation detection of single EBV-transformed cell 1013 using the channel clone normalization method. Single-cell CNV detection of EBV-transformed cell 1013 after channel clone normalization. The y-axis represents the log2 ratios and the x-axis the probe position along the chromosome. The blue line represents the CBS segmentation line. The red region represents the deletion and the green region represents the duplication called by CGHcall. [file gb-2011-12-8-r80-S17.PDF]

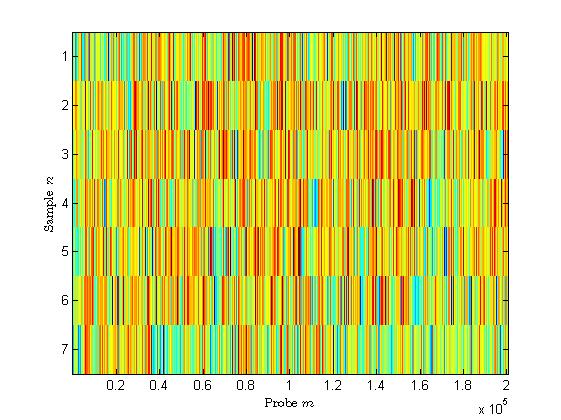

Supplement: Additional file 18 — Figure S18 - genome-wide copy number variation detection of single EBV-transformed cells using the GADA algorithm. Single-cell CNV detection of all seven EBV-transformed cells. Each row represents the profile of one EBV-transformed cell and each column represents one probe across all the EBV-transformed samples. Different colors in the profile represent the breakpoints of single-cell CNVs detected by GADA. [file gb-2011-12-8-r80-S18.JPEG]
